# Convergence and Divergence in the Evolution of Cat Skulls: Temporal and Spatial Patterns of Morphological Diversity

**DOI:** 10.1371/journal.pone.0039752

**Published:** 2012-07-06

**Authors:** Manabu Sakamoto, Marcello Ruta

**Affiliations:** School of Earth Sciences, University of Bristol, Bristol, United Kingdom; University College London, United Kingdom

## Abstract

**Background:**

Studies of biological shape evolution are greatly enhanced when framed in a phylogenetic perspective. Inclusion of fossils amplifies the scope of macroevolutionary research, offers a deep-time perspective on tempo and mode of radiations, and elucidates life-trait changes. We explore the evolution of skull shape in felids (cats) through morphometric analyses of linear variables, phylogenetic comparative methods, and a new cladistic study of saber-toothed cats.

**Methodology/Principal Findings:**

A new phylogenetic analysis supports the monophyly of saber-toothed cats (Machairodontinae) exclusive of Felinae and some basal felids, but does not support the monophyly of various saber-toothed tribes and genera. We quantified skull shape variation in 34 extant and 18 extinct species using size-adjusted linear variables. These distinguish taxonomic group membership with high accuracy. Patterns of morphospace occupation are consistent with previous analyses, for example, in showing a size gradient along the primary axis of shape variation and a separation between large and small-medium cats. By combining the new phylogeny with a molecular tree of extant Felinae, we built a chronophylomorphospace (a phylogeny superimposed onto a two-dimensional morphospace through time). The evolutionary history of cats was characterized by two major episodes of morphological divergence, one marking the separation between saber-toothed and modern cats, the other marking the split between large and small-medium cats.

**Conclusions/Significance:**

Ancestors of large cats in the ‘Panthera’ lineage tend to occupy, at a much later stage, morphospace regions previously occupied by saber-toothed cats. The latter radiated out into new morphospace regions peripheral to those of extant large cats. The separation between large and small-medium cats was marked by considerable morphologically divergent trajectories early in feline evolution. A chronophylomorphospace has wider applications in reconstructing temporal transitions across two-dimensional trait spaces, can be used in ecophenotypical and functional diversity studies, and may reveal novel patterns of morphospace occupation.

## Introduction

Patterns of convergence and divergence of biological shape – both in time and throughout the range of theoretical or realized morphotypes – are key to understanding the dynamics of clade evolution. To this end, a firm phylogenetic framework ensures that convergence is distinguished from morphological similarity due to shared evolutionary history; and that morphological dissimilarities among closely related taxa can be evaluated in terms of evolutionary time separating those taxa.

Cats (Carnivora; Felidae) are excellent model organisms for macroevolutionary analyses of morphological shape diversification. Their relatively recent origin (∼10 million years ago [Ma] for extant Felinae [1] and ∼28.5–35 Ma for Felidae [2]) allows us to investigate patterns of constraint, convergence, and divergence in a successful group of predatory mammals. Extant cats consist of 36 to 41 species assigned to eight genotypic lineages in the subfamily Felinae [1]–[4]. The extinct Machairodontinae, including the popular saber-toothed cats, are generally regarded as the phylogenetically closest relatives to Felinae [5]. The adaptations of cats to hypercarnivory, coupled with their rapid speciation and relatively recent evolutionary origin, explain in part their morphological conservatism [3], particularly evident in the skull. The evolution of skull form and function in fossil and living cats has been subjected to considerable scrutiny [6]–[14], and phylogenetic thinking has informed the interpretation of major patterns of shape change. Several works that considered phylogeny [11]–[14] addressed phylogenetic correction of variance in correlation coefficients [15] linking shape to functional and ecological indices. However, the application of explicit, quantitative phylogenetic comparative methods to the study of felid cranial shape has not been undertaken. Here, we examine in detail patterns of convergence and divergence in skull shape for the majority of extant felines and a cross-section of the best-known machairodontines, using combined morphometric, phylogenetic, and disparity analyses.

Our major goal is to reconstruct temporal transitions in patterns of morphospace occupation. A proper understanding of these transitions benefits greatly from the use of phylogenetic information. To this purpose, we introduce a novel simple method to visualize morphological diversity changes in the evolutionary history of the group. This method – which we term ‘chronophylomorphospace’ (CPMS hereafter) – plots the positions of reconstructed ancestors both in morphospace and through time using a known phylogeny. As an extension and improvement of the phylomorphospace approach [16]–[22], this new method can be applied to a broad range of studies that combine phylogeny and morphospace analyses. Because it takes into account divergence time of estimated ancestral morphotypes, the CPMS allows us to track both phylogenetic and temporal routes through which cats’ ecophenotypical variety was attained.

## Materials and Methods

### Group Delimitations

For extant felid species, we follow the taxonomy of Werdelin et al. [2], based on the molecular tree of Johnson et al. [1]. As a convention, the eight genotypic lineages identified by Johnson et al. [1] were treated as having equal taxonomic rank to the three fossil lineages of saber-toothed cats used here, which we term the ‘Metailurus’, ‘Homotherium’, and ‘Smilodon’ lineages. These three lineages are commonly referred to as the tribes Homotheriini, Metailurini, and Smilodontini, respectively [2], [23]. All saber-toothed felids were placed in the subfamily Machairodontinae, whereas all extant felids (including the ‘Panthera’ lineage cats) were included in the subfamily Felinae [23]. Although subspecies assignments were recorded where information was available, the operational taxonomic units (OTUs) were all considered at the species level. As the specific status of some fossil specimens (e.g., F:AM 62192) is uncertain, they were treated as separate OTUs. This approach offers a partial, independent test of the phylogenetic placement of these specimens. Lineage memberships for each OTU is listed in Table S1.

### Felid Phylogeny

To investigate the covariance in morphospace occupation due to inherited phylogenetic history, and to track patterns of morphological evolution leading to reconstructed morphospace occupancy, we used the molecular tree from Johnson et al. [1] to which we grafted a novel set of relationships for fossil species.

**Figure 1:**
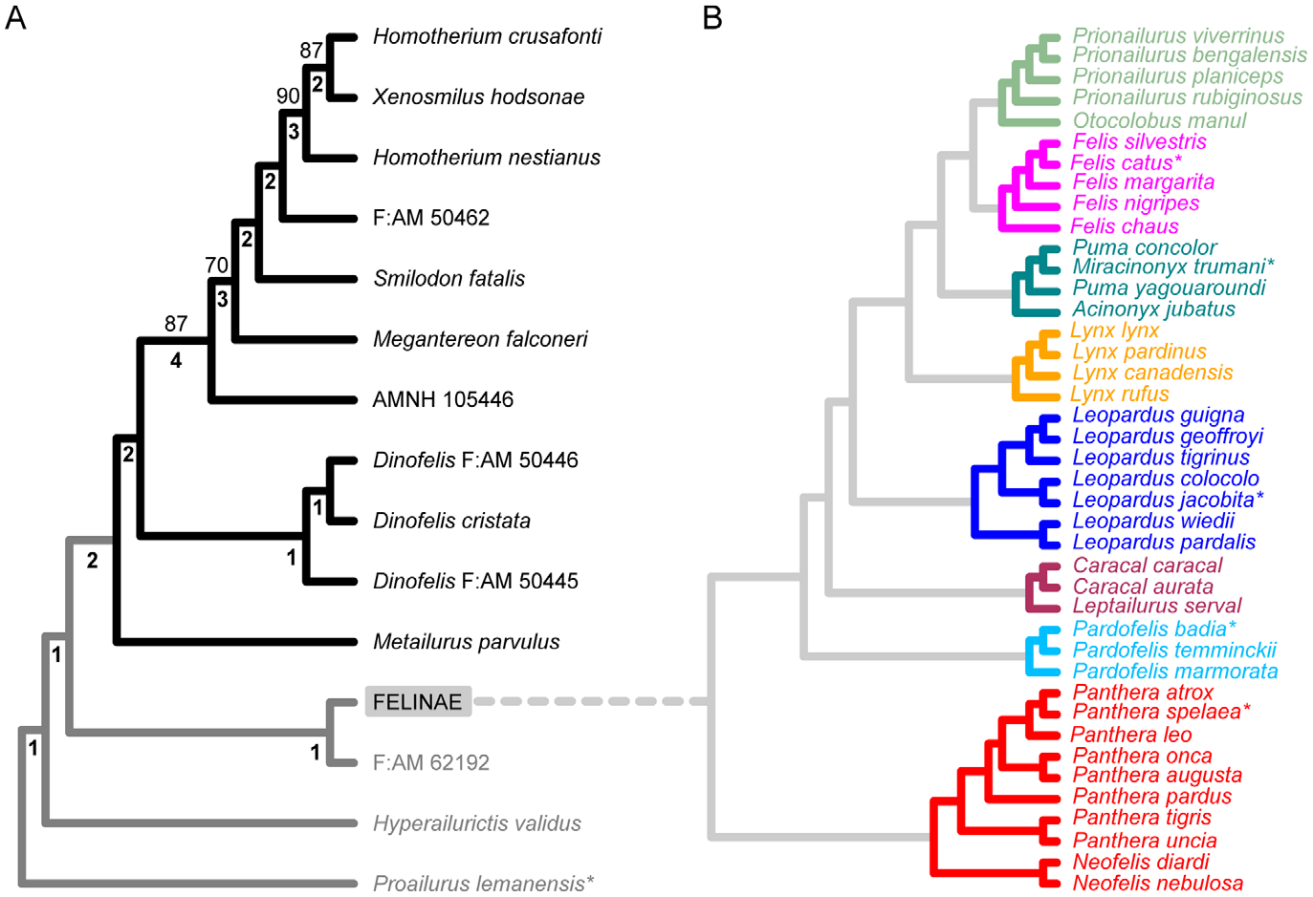
Phylogeny of the Felidae. (A) Single most parsimonious tree from the cladistic analysis, with *Proailurus lemanensis* as the outgroup. Felinae is treated as a single operational taxonomic unit (OTU) in the analysis, but is replaced with the topology in (B) for phylogenetic comparative analyses. Bootstrap percent support values and decay index values (in bold) are shown above and below each node respectively. (B) Internal relationships of Felinae based on [1], with extinct taxa inserted into relevant positions [27]–[29]. Asterisks denote taxa not included in the morphometric analysis, but employed in the phylogeny for dating nodes. Felid lineages are color-coded as follows: black, Machairodontinae; red, ‘Panthera’; sky blue, ‘Bay Cat’; maroon, ‘Caracal’; blue, ‘Ocelot’; orange, ‘Lynx’; green, ‘Puma’; pink, ‘Domestic Cat’; light green, ‘Leopard Cat’.

For the first time, we analysed simultaneously the relationships of basal felids (*Proailurus lemanensis*, *Hyperailurictis validus*, and F:AM 62192), Felinae, and Machairodontinae using a maximum parsimony analysis of 44 discrete characters coded in 14 fossil taxa (see Table S2 for notes on specimens used) to which we added Felinae as a supraspecific OTU (Table S3); 18 of these characters are after Salesa et al. [24] (Text S1; see Table S3 for the character matrix). Tree searches were conducted in TNT [25] and PAUP* [26] with *Proailurus lemanensis* selected as an outgroup. In PAUP*, after an initial heuristic search (5000 random stepwise addition sequences followed by tree bisection-reconnection branch-swapping searching, but holding only one tree in memory at any one time), we searched with the option of unlimited maximum number of trees, swapping branches of the trees in memory from the previous run. These searches resulted in a single most parsimonious tree (MPT; Fig. 1A). Identical options were selected in the traditional search settings of TNT. We used a large number of replicates (e.g. 99999, or as much as the memory permits) in each case to ensure adequate coverage of tree space.

**Figure 2:**
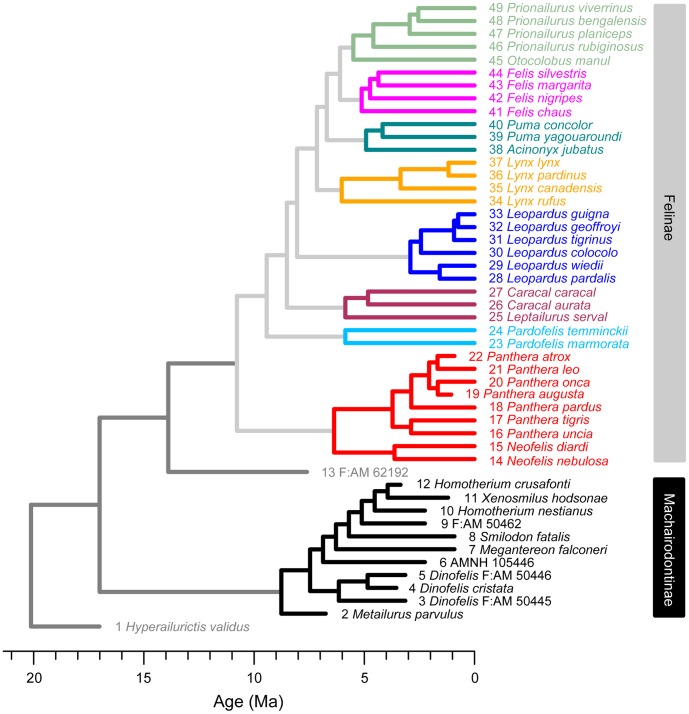
Scaled, pruned composite tree used in phylogenetic comparative methods. The OTU, Felinae, in the single MPT (Fig. 1A) was replaced with the whole tree of Johnson et al. [1] and the branches were scaled following Sakamoto et al. [6]. Extant nodes were dated using either first occurrence or molecular divergence dates, whichever is the older, and terminal branches were extended to their last occurrence dates (i.e. modern time). Since temporal ranges of fossil taxa often have large margins of uncertainties (e.g., Pleistocene: 1.81-0.0117 Ma), the midpoint value of the age range was used to date each node and terminal fossil branches were not extended to their younger limit of the age range. Taxa not represented in the morphometric data were pruned from the scaled, composite tree. Color codings are as in Fig. 1.

Subsequently, we replaced the Felinae OTU in the MPT with the entire tree from Johnson et al. [1] (Fig. 1B) to produce a composite phylogeny (Fig. 2). The rationale behind this approach is that an all-encompassing species-level phylogeny of living and fossil cats is beyond the scope of the present work, and must await thorough scrutiny of data matrices published so far and a comprehensive revision of both character formulation and character-state delimitations. Three fossil felines, *Panthera atrox*, *P. spelaea*, and *Miracinonyx trumani*, were inserted into their relevant positions according to [27]–[29]. Because the position of *Panthera palaeosinensis* found by [30] relies on a different set of relationships among *Panthera* species from that in Johnson et al. [1], we omitted this fossil species from our analyses. The Pleistocene North American jaguar, *Panthera augusta* (or *P. onca augusta*) [31], [32], was placed as the sister taxon to the jaguar, *P. onca*. These fossil *Panthera* species, *P. atrox*, *P. spelaea*, and *P. augusta*, were treated as separate species-level OTUs rather than as subspecies of *P. leo* (lion) or *P. onca*, to capture in greater detail morphological changes through time.

### Tree Branch Scaling

The branches of the composite phylogeny were scaled to reflect divergence dates between taxa. Branches were scaled either according to node dates estimates in the molecular phylogeny of Johnson et al. [1] or using fossil occurrence dates, whichever yielded the older date for any given node (see reference [6] for a more detailed description). As many extinct taxa have uncertain dates (large stratigraphic range mostly resulting from uncertain dating of the fossil-bearing strata in some localities), midpoint dates of their respective stratigraphic ranges were used (Table S4) [33], [34]. Fossil occurrence dates were compiled from various sources (Table S4) based on locality and age information associated with the specimens. To circumvent the problem of zero length branches (resulting from the dating of internal nodes leading to terminal taxa with identical earliest known occurrences), we followed the protocol expounded by Brusatte et al. [22]. Extant branches were first dated using first occurrence dates (Table S4) and further extended to the current age (i.e., 0 Ma); fossil terminal branches were not extended to their youngest stratigraphic range (but see Text S2 for alternative combinations of dates, Figs. S7 and S8 for scaled trees using these alternative dates, and Fig. S9 for an alternative CPMS plot). In estimating branch lengths, a larger tree is preferable over a smaller tree (i.e., number of taxa representing only those in the morphometric analysis) because additional stratigraphic/divergence information from taxa interspersed amongst those of the smaller tree contributes more accurate age estimates for internal and increasingly more basal nodes. This scaled composite tree was then pruned to reflect the taxonomic sample of the morphometric data (Fig. 2). Similarly, taxa that are not present in the phylogeny (*Dinobastis serus*; ‘*Metailurus*’ IVPP-5679) were discarded from the morphometric data.

### Morphometric Analyses

**Figure 3:**
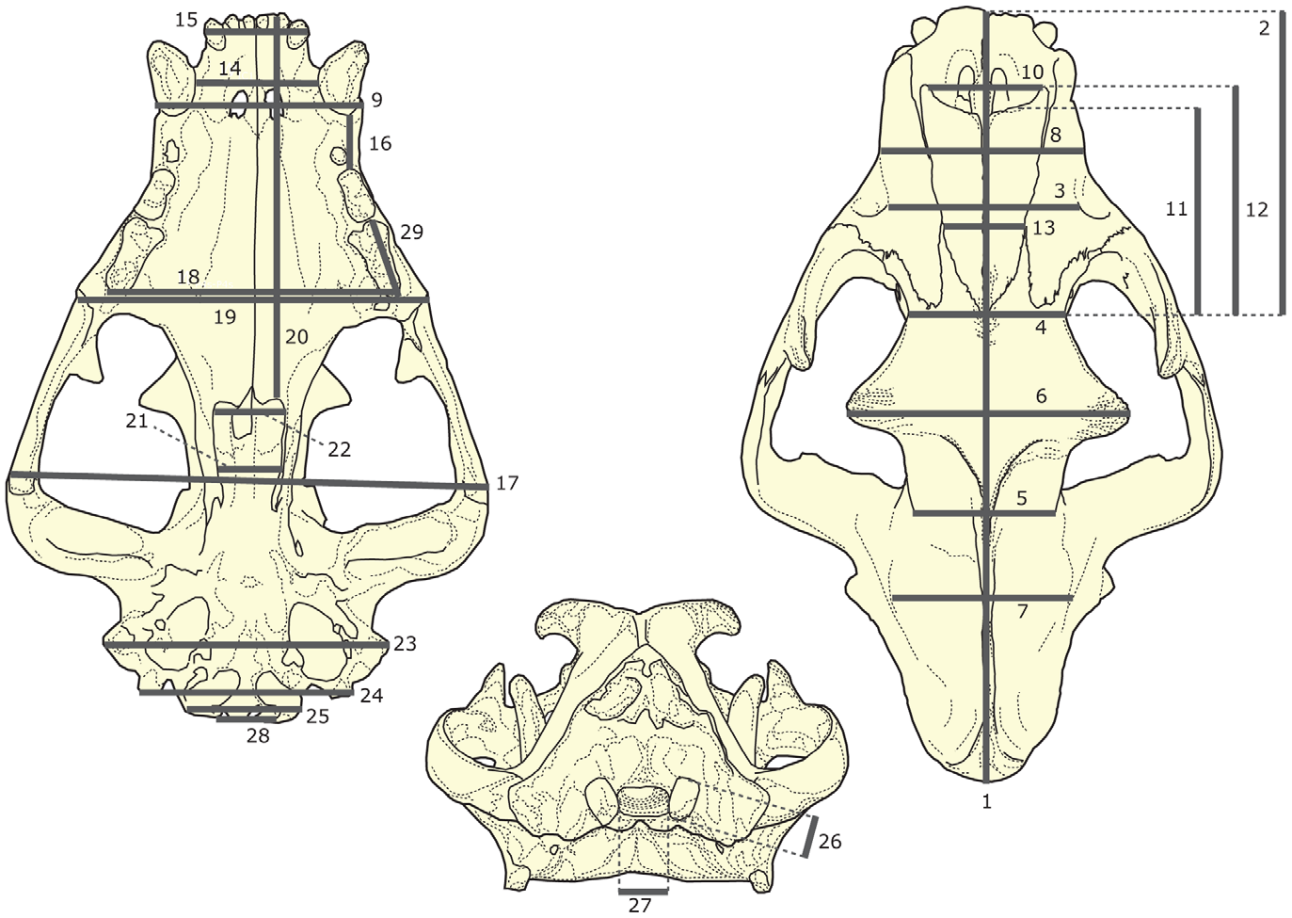
Description of cranial measurements. A skull of a lion is shown with diagrammatic representations of the 29 cranial measurements used in the morphometric analyses. The actual measurements are Euclidean distances between two points on the skull specimen and are not two-dimensional projections as depicted here. 1, *L*_SkT_: total skull length (distance between inion and prosthion). 2, *L*_Face_: face length (distance between prosthion and naso-frontal suture). 3, *W*_iof_: distance between infraorbital foramina. 4, *W*_o_: distance between orbits. 5, *W*_POC_: width across postorbital constriction. 6, *W*_POP_: width across postorbital processes. 7, *W*_BC_: maximum braincase width (greatest distance between lateral margins of braincase). 8, *W*_sn_: snout width (measured at level of snout mid-length). 9, *W*_C1s_: width across the snout (measured between bases of upper canines [C1s]). 10, *W*_NA_: nasal aperture width (measured at rostral projection of nasals). 11, *L*_N_: nasal length (measured parasagittally between naso-frontal suture and dorsal margin of external narial opening). 12, *L*_NT_: total nasal length (measured parasagittally as the distance between the naso-frontal suture and the anteriormost tip of the nasal). 13, *W*_MFS_: width across the nasals (measured between the left and right maxillo-frontal sutures [MFS]). 14, *W*_IC1s_: inter-canine width (measured between the upper canines). 15, *W*_I3s-I3s_: width across the incisor arcade (measured between left and right third upper incisors [I3s]). 16, *L*_C1sP3s_: length of upper ‘diastema’ (measured as the distance between C1s and upper third premolars [P3s]); although species that possess upper second premolars (P2s) do not show a diastema, we regarded their *L*_C1sP3s_ as the topological equivalent of the mandibular diastema. 17, *W*_Sk_: maximum skull width across zygomatic arches. 18, *W*_P4s-P4s_: palate width (measured between labial surfaces of upper fourth premolars [P4s]). 19, *W*_Pal_: palate width (measured posterior to P4s and between lateral surfaces of maxillae). 20, *L*_Pal_: palate length (measured parasagittally as the distance between the prosthion and the posterior extremity of the palatal surface). 21, *W*_PN_: internal width of postnarial opening (measured as the distance between left and right pterygoid flanges). 22, *W*_PN.ant_: internal width of anterior postnarial opening (measured immediately posterior to palate). 23, *W*_MP_: width across mastoid processes. 24, *W*_PocP_: width across paroccipital processes. 25, *W*_OC_: occipital condyle width. 26, *H*_OC_: occipital condyle height. 27, *W*_FM_: internal width (transverse diameter) of foramen magnum. 28, *W*_FMV_: ventral width of foramen magnum (distance between left and right occipital condyles). 29, *L*_P4s_: length of superior fourth premolar or carnassial.

Twenty-nine cranial linear variables (see Fig. 3) were measured in 332 specimens (Table S5) encompassing 34 extant and 18 extinct felid species, covering 37 felines, 13 machairodontines and 2 basal taxa. As the measurements are linear, we adjusted for the effect of size (isometric scaling) by dividing them by their geometric mean for each specimen (Table S6) [35]. The geometric mean (GM) is the *k*th root of the product of the values of the *k* variables for the specimen in question, i.e., (Π*a*_i_)^1/*k*^ where *a*_i_ is the morphometric variable of interest. As GM is in the same unit as the original variables, the resulting ratios are dimensionless. These ratios – sometimes referred to as Mosimann shape variables [9] – have been previously shown to perform better than residuals as size adjusted shape variables [36]. Further, unlike residuals, Mosimann shape variables correct for scaling using information that relates solely to the specimen that is being measured, and do not rely on trends from other individuals.

We conducted a Linear Discriminant Analysis (LDA) to determine the ability of the transformed linear variables to discriminate specimens based on their prior classifications. To this purpose, 330 specimens (excluding *Hyperailurictis validus* and F:AM 62192) were assigned to one of the extant lineages (‘Bay Cat’, ‘Caracal’, ‘Domestic Cat’, ‘Leopard Cat’, ‘Lynx’, ‘Ocelot’, ‘Panthera’, and ‘Puma’) [1], or one of the fossil lineages (‘Homotherium’, ‘Metailurus’, and ‘Smilodon’). Although the phylogenetic analyses above found ‘Metailurus’ and ‘Smilodon’ lineages to be paraphyletic (Fig. 1A), we treated them here as grouping categories for convenience and ease of description. The classification accuracy of the LDA was assessed through a jackknife approach (‘leave-one-out’ cross validation). Specifically, LDA was performed *N* times, where *N* represents the total number of specimens (330 in our case), but excluding one specimen at a time. In each run, the resulting discriminant functions were used to predict the classification of that specimen; this prediction is unbiased by the specimen in question because the discriminant functions are derived following the exclusion of that specimen. Calculations were repeated for each specimen. The overall proportion of specimens that are correctly assigned to prior groups indicates how well the discriminant functions predict classifications of new data. Classification accuracies of each lineage can also elucidate patterns of morphological similarities or distinctiveness between various lineages (Table S7).

A morphospace was built from a multivariate ordination of the size-adjusted variables using Principal Components Analysis (PCA), with all variables scaled to unit variance. Differences between groups (i.e., the separation between lineages or species) in morphospace were evaluated with a non-parametric multivariate analysis of variance (NPMANOVA) in the software PAST v. 2.14 [37], to test the null hypothesis of equality of the variances of the PC scores. Because NPMANOVA is non-parametric, it is appropriate in the absence of information on the distribution of the scores. The test’s *F* statistic and associated level of significance were calculated with 9999 permutations, and adjusted with sequential Bonferroni correction to account for multiple pair-wise comparisons. Comparisons were made among the 11 felid lineages (eight feline lineages plus three machairodontine lineages) on PC1–PC11 axes, and excluding *Hyperailurictis validus* and F:AM 62192, as in LDA.

Size adjustment, LDA and PCA were performed in the R environment for statistical calculations [38]. Note that, because the multivariate analyses were conducted on specimens rather than species, we did not introduce correction for nonindependence among observations. Such procedures (e.g. phylogenetic PCA and phylogenetic LDA) are more appropriate in the case of species-level morphospaces [39], [40].

We did not include any fossil specimens with substantial distortion to cranial proportions (e.g. crushing or shearing) since these specimens tend to fall out at the extremities of morphospace. We did however include three individuals with minor distortions: the *Dinofelis cristata* specimen, M3657; and the two casts of *Megantereon falconeri*. The separation in morphospace between M3657 and the other two ‘*Dinofelis*’ specimens F:AM 50445 and F:AM 50446, but also the separation between the two *M. falconeri* individuals, are relatively high (Fig. S2), but they are overall comparable to the degree of separation of members in a well-sampled, highly disparate taxon (e.g. *Puma concolor*, *Leopardus pardalis* and some *Panthera* species). Thus it is not possible to distinguish the difference between high within-taxon morphological variability due to distortion from that due to natural variability.

### Phylogenetic Signal in Morphospace

In order to investigate the relationship between phylogeny and morphospace occupation, phylogenetic comparative methods (PCM) were employed. PCs were tested for the presence of phylogenetic signal using two methods: phylogenetic eigenvector regression (PVR) [41] and Blomberg’s *K* statistic [42]. PVR is a type of multiple linear regression where the variable[s] of interest represent the response variable(s) (i.e., the PC scores) and the phylogenetic eigenvectors (obtained from a principal coordinates [PCO] analysis of pair-wise Euclidean distances built from branch lengths) represent the predictor variables (specifically, the PCO scores). The pruned tree was subjected to a PCO analysis. The appropriate number of PCO axes was determined by an arbitrary cut-off of 95% cumulative variance. The first 23 PCO axes satisfied this cut-off threshold, and were thus retained and used in PVR. Species-mean values of morphometric PCs were computed for the taxa represented in the phylogeny. A matrix of the first 11 PC axes were treated as the response variable matrix. A multivariate form of PVR (MPVR) [43] was conducted in R using a script written by the senior author in order to assess the proportion of variance explained by the regression model and to determine its significance.

An alternative way of detecting phylogenetic signal is the method of Blomberg et al. [42] implemented in R in the picante package [44]. This method uses phylogenetically independent contrasts (PIC; e.g., see [45]) to compare the variances of the contrasts computed for a given variable on a particular tree topology with those computed from random permutations of that variable across the same tree (i.e., randomly reshuffling the values of the variable amongst the OTUs while keeping the tree topology constant). If the variances in the contrasts for the data in the observed phylogenetic positions are lower than those from the permutations, then there is a significant phylogenetic signal in that data [42]. Blomberg’s *K* statistic [42], [44] quantifies the strength of this phylogenetic signal. If *K* <1, then closely related OTUs have values that are less similar than expected under a Brownian model of evolution (such as a model of evolution with adaptive constraints), while a *K* >1 would indicate that closely related OTUs have values more similar than expected (strong phylogenetic signal) [42].

### Chronophylomorphospace

Patterns of morphospace occupation across phylogeny can be investigated by reconstructing a phylomorphospace [16]–[22]. This typically involves ancestral character estimation of morphospace coordinate values for each internal tree node, using squared change parsimony [46] or maximum likelihood (ML) [47], among other methods, and the reconstructed ancestral values are plotted onto the two-dimensional morphospace together with the OTUs. Internal nodes are then connected according to phylogeny structure. This approach results in a two-dimensional projection of phylogeny onto morphospace. However, a phylogeny does not represent exclusively the interrelationships of the OTUs; it also includes data on their temporal divergence. Thus, a two-dimensional phylomorphospace accounts for the first aspect of phylogeny, but does not necessarily faithfully represent the second aspect. A more complete representation of the changes in morphospace occupation in different groups and throughout the sequence of branching events involves the inclusion of a time component (i.e., branch lengths). This can be achieved by adding a time axis as a third dimension to the two-dimensional phylomorphospace. The X-Y coordinates of ancestor values are reconstructed as usual from terminal values and from a scaled phylogeny using the ape R library [48], and are subsequently plotted along the temporal Z-axis according to their positions in time, such as is calculated from stratigraphic data of OTUs and from branch length information (one-dimensional or single-trait implementations have been presented previously [6]; but see also Figs. S5, S6). A new R function, chronoPTS2D, was written to plot an interactive three-dimensional CPMS (Video S1), utilizing the rgl library [49], which allows for spinning, zooming in and out, and generating animations. The function chronoPTS2D can be implemented on other examples of two-dimensional trait space, such as function space [43], and are available upon request.

### Institutional Abbreviations

AMNH, American Museum of Natural History, New York, USA; BCMAG, Bristol City Museum and Art Gallery, Bristol, UK; BMNH, Natural History Museum, London, UK; BRSUG, School of Earth Sciences, University of Bristol, Bristol, UK; BRSUVA, Department of Anatomy, School of Veterinary Science, University of Bristol, Bristol, UK; CM, Carnegie Museum of Natural History, Pittsburgh, USA; F:AM, Frick Collection, American Museum of Natural History, New York, USA; HM-NH, Natural History, Horniman Museum and Gardens, London, UK; KPM, Kanagawa Prefectural Museum of Natural History, Odawara, Kanagawa, Japan; NSMT, National Science Museum, Tokyo, Japan; PMU, Paleontological Museum of Uppsala University; RCSOM, Hunterian Museum, Royal College of Surgeons, London, UK. Other abbreviations refer to high-fidelity skull replicas of specimens in official repositories (e.g., Institute of Vertebrate Paleontology and Paleoanthropology, Beijing, China), or to commercially available casts (e.g., Bone Clones; Skulls Unlimited). Replicas and casts were used where original specimens could not be accessed. Access to each collection was granted by the collection manager of the relevant institution.

## Results

The tree of fossil cats (plus Felinae as an OTU) is 87 steps long, with an ensemble consistency index of 0.667 (excluding uninformative characters), an ensemble retention index of 0.776, and an ensemble rescaled consistency index of 0.526. Bootstrap percentage support (10,000 replicates; fast stepwise addition sequence) is low to moderate, and some branches are collapsed in a 50% majority-rule consensus topology built from all bootstrap replicates (Fig. 1A). With reference to the most parsimonious tree, moderate support is assigned to the node subtending all saber-toothed cats more apical than AMNH 105446 (70%). Slightly higher support is assigned to the node linking AMNH 105446 to more apical saber-toothed cats (87%), to the clade formed by *Homotherium nestianus*, *Xenosmilus hodsonae*, and *Homotherium crusafonti* (90%), and to the clade formed by the latter two species (87%). Decay index values (or Bremer support: that is, number of additional steps required to collapse a tree node) are distributed as follows: four extra steps are required to collapse the AMNH 105446-*Megantereon-Smilodon-*F:AM 50462*-Homotherium-Xenosmilus* clade; three extra steps are required to collapse the *Megantereon-Smilodon-*F:AM 50462*-Homotherium-Xenosmilus* clade, and the *Homotherium-Xenosmilus* clade; two extra steps are required to collapse most internal nodes in the machairodontines (except the *Dinofelis* clade nodes, which collapse at just one extra step); all remaining nodes collapse at one additional step. In the new phylogeny (Fig. 1A), some novel patterns of relationship emerge, including: 1, the retrieval of the ‘Metailurus’ and ‘Smilodon’ lineages as grade groups rather than clades, contrary to previous hypotheses [2], [23]; and 2, the paraphyly of *Homotherium*.

The LDA of the 29 transformed linear variables shows their remarkable ability to separate specimens according to their prior classification (i.e., specimen attribution to each of the 11 cat lineages). Classification accuracy (i.e., proportions of correctly vs. incorrectly attributed specimens) based on successive specimen deletion (jackknife) is generally high (overall correct classification rate  = 88.2%), and ranges from 64% in the ‘Puma’ lineage to 100% in the ‘Smilodon’ lineage (Table S7). The low accuracy in predicting ‘Puma’ lineage members is unsurprising as this lineage encompasses three disparate morphotypes, namely the unique cheetah (*Acinonyx jubatus*), the panther-like puma (*Puma concolor*), and the ocelot-like jaguarundi (*P. yagouaroundi*). Individual classifications and posterior probabilities show that while cheetah and most puma specimens are correctly assigned to the ‘Puma’ lineage, all jaguarundi specimens were incorrectly assigned either to the ‘Bay Cat’, the ‘Caracal’, or the ‘Ocelot’ lineages (Table S8).

**Figure 4:**
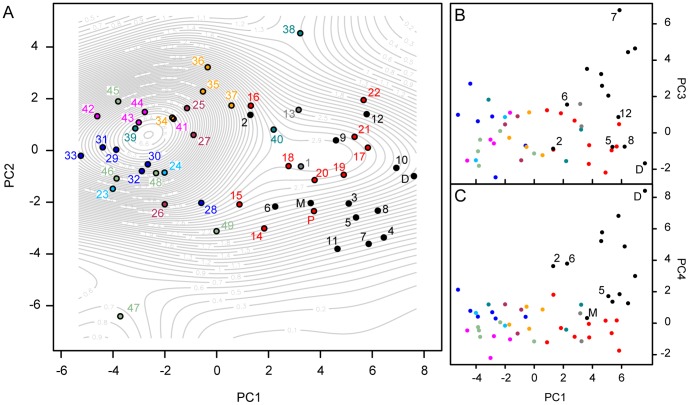
Two-dimensional morphospace plots of species centroids (color-coded spheres) based upon PCA of 29 size-adjusted cranial linear variables in 332 specimens of extant and fossil felids. (A) Two-dimensional morphospace plot delimited by PC1 and PC2, with contour lines showing the spatial density of the specimen-level distribution. (B) Two-dimensional morphospace plot delimited by PC1 and PC3. (C) Two-dimensional morphospace plot delimited by PC1 and PC4. Taxa labels are the same as in Fig. 2, except as follows: D, *Dinobastis serus*; M, *Metailurus* IVPP-5679; P, *Panthera paleosinensis*; these three taxa were not included in the phylogeny. Color codings are as in Fig. 2.

A PCA of the 29 transformed linear variables resulted in 11 Principal Components (PC) axes that account for more than 90% of the total morphological variance (see Table S9 for individual scores along all axes). The PC1 axis accounts for 38% of the total variance, while PC2, PC3, and PC4 account for 14%, 9.6%, and 7.6%, respectively (see Fig. S1 for PCA loadings on these four PC axes). The overall separation among lineages in morphospace is significant (NPMANOVA *F*  = 40.1; *p*  = 0.0001; see post-hoc pairwise comparisons in Table S10), and the distribution density of specimens is mostly unimodal (see profile of contour lines; Fig. 4A), though there is a distinct separation between large and small-medium cats (Fig. 4A; Fig. S2).

Three major features emerge from the two-dimensional morphospace plot delimited by the PC1 and PC2 axes (Fig. 4A; Fig. S2). The first feature is the presence of a size gradient along PC1 (larger species towards positive PC1 scores). As the morphological variables are Mosimann shape variables and are adjusted for isometric scaling, this size-associated trend in PC1 is interpreted as most likely reflecting some true allometric pattern in shape change with size. This allometric pattern is associated with an increase in facial length (*L*_face_), palate length (*L*_pal_), snout width at the canines (*W*_C1s_), nasal width at the fronto-maxillary suture (*W*_MFS_), nostrils width (*W*_NA_), nasal length (*L*_Na_, *L*_NaT_), width across the incisor arcade (*W*_I3s-I3s_), mid snout width (*W*_sn_), and interorbital width (*W*_O_), and with a decrease in the postorbital processes width (*W*_POP_, *W*_POC_), braincase width (*W*_BC_), foramen magnum width (*W*_FM_, *W*_FMV_), and occipital condylar width (*W*_OC_).

The second feature of the morphospace plot is the separation of mid-sized cats into two distinct regions along PC2 (Fig. 4A; Fig. S2). PC2 is primarily associated with a decrease in skull length (*L*_SkT_), palate length (*L*_pal_), mastoid process width (*W*_MP_), paroccipital process width (*W*_PocP_), and P4 length (*L*_P4s_), on the one hand, and with an increase in postnarial width (*W*_PN_, *W*_PN.ant_), snout width at the infraorbital foramina (*W*_iof_), interorbital width (*W*_o_), and postorbital constriction width (*W*_POC_) on the other. Thus, taxa that score positively along PC2 tend to have short stout skulls while those that score negatively tend to have long narrow skulls.

The third feature of the morphospace plot is the occurrence of both unique (i.e., outliers) and convergent morphologies in various phylogenetically distinct taxa. The cheetah (*Acinonyx jubatus*) and the flat-headed cat (*Prionailurus planiceps*) offer examples of outliers, as they plot out in diametrically opposite morphospace areas. Eurasian lynx (*Lynx lynx*), the snow leopard (*Panthera uncia*), and the extinct *Metailurus parvulus* – all exhibiting ‘bulbous’ skulls and wide foreheads – offer examples of convergent skull morphologies. Thus, although these three taxa are phylogenetically distinct, they are close to one another in morphospace.

In the right-hand side of the plot, large *Panthera* species (the big cats) plot out close to various large saber-toothed cats (e.g.; *Homotherium*; some *Dinofelis*). However, some of the largest saber-toothed cats, including *Smilodon* and *Xenosmilus*, occur more peripherally at one extreme of the range of variation of large felids as a whole. Thus, saber-toothed cats show a degree of cranial shape diversity that is unmatched by that of the extant large cats. The basal felid, *Hyperailurictis validus*, is phenetically very similar to the leopard (*Panthera pardus*). Machairodontine taxa are further separated from the feline taxa along PC3 (Fig. 4B; Fig. S3) but more prominently along PC4 (Fig. 4C; Fig. S4). Machairodontines (including the feline-like *Metailurus parvulus* and *Dinofelis*) score positively along PC4 compared to felines with similar PC1 scores (i.e., of similar sizes). Positive PC4 scores are associated with increases in *W*_o_, *W*_I3s-I3s_, and *L*_P4s_, and decreases in *L*_N_, *L*_NT_, *L*_C1sP3s_, and *W*_Sk_, thus reflecting a widening of the snout, enlargement of P4, shortening of the nasals, reduction of the upper diastema, and narrowing of the skull. All these features have been traditionally used to distinguish machairodontines from felines. Furthermore, they add to lower dental and postcranial data that also ally *Dinofelis* to machairodontines [50].

**Figure 5:**
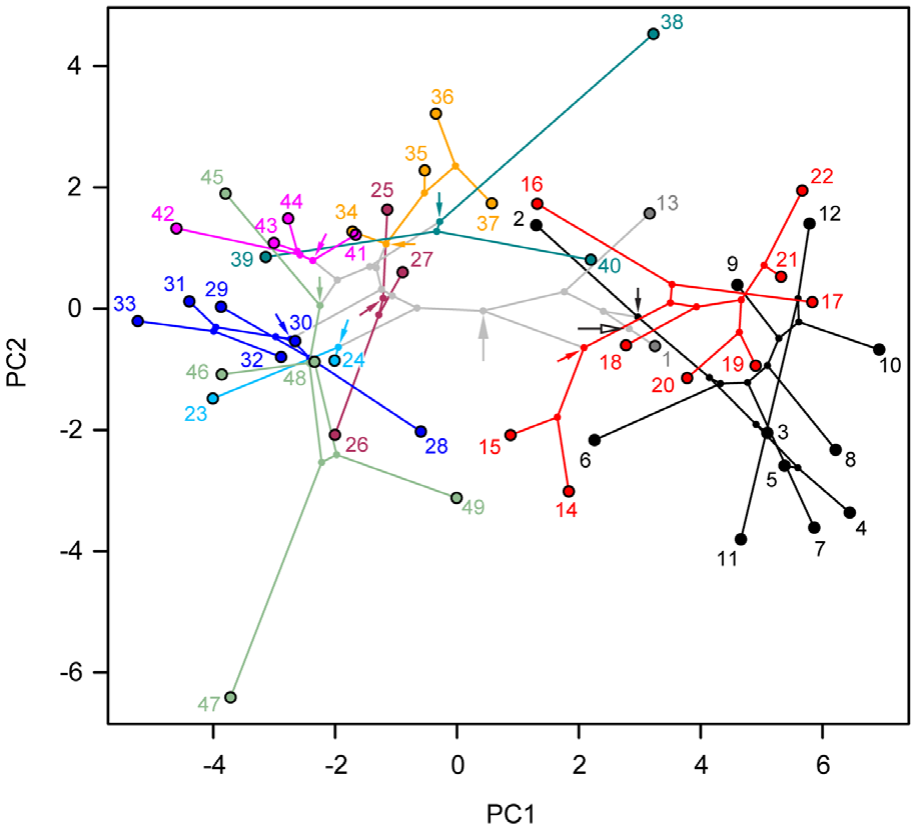
Two-dimensional phylomorphospace plot. The felid phylogeny (Fig. 2) was superimposed onto the two-dimensional morphospace delimited by the first two PC axes (Fig. 4A) using maximum likelihood ancestor character estimation. Arrows indicate ancestral nodes for clades of interest and color-coded as in Fig. 2 but also: open, Felidae; and grey, Felinae. Numbers and colors are as in Fig. 2.

Using MPVR, we found a significant correlation (*p*  = 3.25×10^−13^) between the morphospace matrix and the phylogeny matrix, with about 78% of variance in morphospace and, separately, about 90%, 69%, 62% and 86% of variance in PC1, PC2, PC3 and PC4, respectively, explained by phylogeny. Blomberg’s test shows phylogenetic signal to be significant and strong in PC1 (*K*  = 1.24; *p*  = 1×10^−4^), significant but weak in PC2 (*K*  = 0.305; *p*  = 5.50×10^−3^), not significant in PC3 (*K*  = 0.246; *p*  = 0.128) and significant but relatively weak in PC4 (*K*  = 0.809; *p*  = 1×10^−4^). This overall strong phylogenetic signal is evident in the phylomorphospace plot (Fig. 5) where branch overlap within lineages is minimal. The ancestral position of Felidae is reconstructed proximally to the modern leopard (*Panthera pardus*), which therefore provides a suitable modern analogue for the ancestral felid skull morphology.

**Figure 6:**
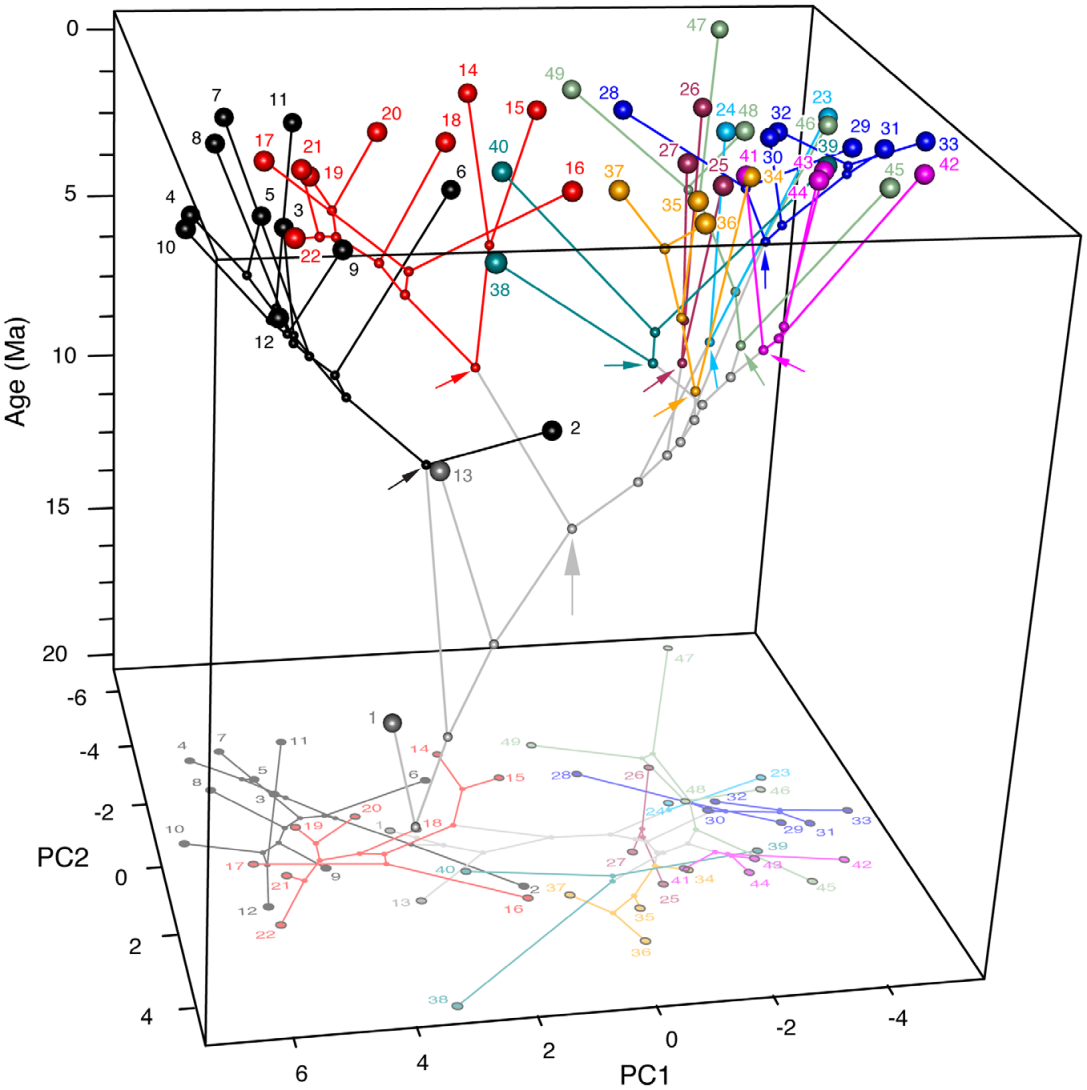
Chronophylomorphospace of Felidae. The transition of two-dimensional morphospace occupation through time can be visualised as a chronophylomorphospace plot. Positions in morphospace of ancestors were estimated using maximum likelihood and a composite phylogeny of Felidae with scaled branch lengths (Fig. 2). Two-dimensional coordinates of ancestors and terminals were then plotted against time as the third axis. Color-coded arrows point to the latest common ancestor of each lineage. The grey arrow points to the last common ancestor of Felinae. The drop-down shadow shows a planar projection of the chronophylomorphospace on the two-dimensional morphospace area delimited by the PC1 and PC2 axes. Numbers and colors are as in Fig. 2.

Our CPMS (Fig. 6; Video S1) adds a temporal axis to the standard two-dimensional phylomorphospace plot (Fig. 5), and reveals three notable patterns. The first is at the base of Felidae. Here, *Hyperailurictis validus* and F:AM 62192 are both morphologically distinct and occupy separate phylogenetic positions (Fig. 1A). *Hyperailurictis validus* diverges very little from the reconstructed position of the ancestral felid node in the two-dimensional morphospace. Unlike *Hyperailurictis validus*, F:AM 62192 has a unique evolutionary trajectory, in that it diverges considerably from its ancestral node, and plots out in a morphospace region that is subsequently convergently occupied by the modern puma (Fig. 5).

A second remarkable pattern is the early and conspicuous divergence between machairodontines and felines. Successive internal nodes of the machairodontine phylogeny plot out along a less steep trajectory than internal nodes leading to major feline lineages; this trajectory appears to ‘spiral out’ towards the diversity of the largest members of the saber-toothed cats. The ancestral nodes in the machairodontine portion of the phylogeny overlap with the ancestral nodes of the large *Panthera* cats in the two-dimensional projection of phylomorphospace (Fig. 5), but are chronologically well separated from those (Fig. 6; Video S1).

Finally, on the feline portion of the tree, we remark a second early and conspicuous divergence, between the clade of large cats (‘Panthera’ lineage) and the clade of small-medium cats (all other lineages). This divergence is also characterized by almost complete non-overlap in the history of these two clades. The small-medium cats appear to be fairly constrained in their patterns of ancestral morphospace occupation. In particular, whereas the respective basal nodes of the modern small-medium cat lineages tend to occur far apart, the deeper internal nodes from which these basal nodes diverge are more closely spaced. The CPMS further highlights the remarkable outlying position of the flat-headed cat and the cheetah, due to their early divergence from their respective ancestral nodes.

## Discussion

While molecular phylogenetics have recently advanced our understanding of the relationships amongst modern cats, the mutual phylogenetic positions of Felinae, Machairodontinae, and various early felids (e.g., *Proailurus*, *Hyperailurictis*, F:AM 62192) have received less attention. A previous analysis of the interrelationship of North American ‘*Pseudaelurus*’ taxa [51] recovered this genus as a paraphyletic ‘grade’ along the stem of Felinae (represented by *Lynx canadensis* and *Puma concolor*), but the analysis in question did not include any machairodontine taxa. Other authors have hypothesized that various *Pseudaelurus*-like taxa could be ‘ancestral’ to both ‘conical’-toothed cats (Felinae) and saber-toothed cats (Machairodontinae) [2], [23]. Our analysis shows that at least one *Pseudaelurus*-like taxon (F:AM 62192) is more closely related to Felinae than it is to any other felid species (including *Hyperailurictis validus* and all machairodontines). Thus our results find *Pseudaelurus*-like taxa to form a polyphyletic assemblage, as proposed by [2], [23], although since we have not analyzed those *Pseudaelurus* species previously hypothesized to be on the machairodontine stem (such as *P. quadridentatus* [2], [23]), our new phylogeny cannot be used to test this hypothesis. Further, it is also possible that F:AM 62192 is actually a member of Felinae, but since we have treated the latter as a supraspecific OTU, further analysis including numerous feline taxa is necessary to resolve this.

The detailed species-level interrelationships of machairodontines have also received limited treatment within a cladistic framework, certainly in terms of number of species considered [24], [52]. To the best of our knowledge, our analysis is the first to include all major machairodontine lineages (in particular the ‘Metailurus’ lineage cats) in a single cladistic matrix, and offers a preliminary numerical test of phylogenetic hypotheses put forward by previous authors [2], [23]. We recover a monophyletic Machairodontinae, with the three major saber-tooth lineages – ‘Metailurus’, ‘Smilodon’ and ‘Homotherium’ –, being more closely related to each other than to other felids (Felinae, *Hyperailurictis*, and *Proailurus*). However, the shape of the machairodontine phylogeny differs from previous hypotheses in two major aspects.

First, the ‘Metailurus’ lineage, commonly referred to as Metailurini [2], [23], does not form a monophyletic group, and emerges instead as a grade group. Within the latter, *Dinofelis* forms a monophyletic genus, with *D. cristata* as the sister taxon to F:AM 50446. F:AM 50445 and 50446 are from the same locality (Ruscinian – Villafranchian of Niu Wa Kou, Shanxi, China), and their separation in the phylogeny is exclusively due to different states of character 34, concerning the shape of the naso-frontal suture.

Second, the ‘Smilodon’ lineage, commonly referred to as Smilodontini, and including the genera *Megantereon* and *Smilodon*, is similarly recovered as a paraphyletic group instead of a clade.

Failure to retrieve support for the monophyly of two of the three major clades in this analysis may be due in part to limited character and taxon sample size, and future rigorous analyses based on expanded character and taxon sets are likely to cast new light on the nature of the conflict in published phylogenetic hypotheses. While we acknowledge the limitations of the current analysis of fossil taxa, we believe it is a small step forward towards future, more comprehensive undertakings; by far the greatest challenge is a proper evaluation of character polarity of Felidae as a whole in light of simultaneous inclusion of fossil and extant taxa.

While the overall distribution of taxa in morphospace and patterns of shape change are consistent with previous analyses [7]–[14], the convergence of the machairodontine, *Metailurus parvulus*, with the Eurasian lynx and with the snow leopard contrasts with a recent study [11] in which *Metailurus* plots out with *Panthera* species other than the snow leopard. This discrepancy may be due to the fact that the analyses in [11] used lateral projections of the skull, whilst our work also considers variables that relate to skull width. Furthermore, *Metailurus* resembles the snow leopard in limb robustness, and the Eurasian lynx in limb proportions [53]. These similarities suggest that *Metailurus* presumably partly occupied similar ecological roles to these extant taxa in the open woodlands of Greece or the subarid steppes of China, where its remains have been found [54], [55]. This suggestion is entirely speculative and requires further testing in light of other character correlates and detailed palaeoecological analyses.

On a purely methodological note, our study demonstrates the effectiveness of an adequate sample of morphological descriptors in morphometric analyses. For example, the machairodontine, *Dinofelis*, was found to be phenetically close to *Panthera* in [9], [11]. However, our analyses place it in proximity to other large machairodontines, based upon its skull proportions, its robust and wide snout, and its elongate face.

Overall, size-adjusted linear measurements summarize a large amount of morphological variation and produce multivariate ordination results that are comparable to those from landmark-based geometric morphometric analyses. Further, LDA reveals that they can accurately predict lineage memberships. For these reasons, size-adjusted linear variables (and by extension traditional morphometrics) remain a powerful tool in studies of biological shape that complements the thrust and potentials of geometric morphometrics.

Phylogeny provides a useful framework for mapping trait evolution [6], [16]–[22], [43]. Phylogeny reconstruction embodies, among others, two aspects of evolutionary history: 1, the interrelationship amongst OTUs; and 2, the temporal scale of branching events. A phylogeny mapped onto morphospace (phylomorphospace; e.g., see [21]) only incorporates the first aspect of phylogeny but lacks temporal information. A more accurate way of depicting the evolutionary dynamics of morphospace occupation across phylogeny is to include temporal data. Our CPMS (Fig. 6; Video S1) accomplishes this by visualizing changes in morphospace occupation among various lineages and through time.

The CPMS reconstructs the early history of cat morphospace occupation as being relatively restricted to the regions of morphospace where medium-large sized cats plot out. This could potentially be due to lack of fossils of small-sized cats from this time (the oldest unequivocal feline, *Pristifelis attica* is known from around 5–9 Ma [2], [56]), and it is possible that future discoveries will fill this gap. The remarkable ‘burst’ of morphospace occupation does not occur until slightly later (at around 10 Ma) when the respective ancestors of large-bodied taxa (Machairodontinae and ‘Panthera’ lineage cats) diverge from the small/medium-bodied (non-‘Panthera’ lineage). While the full extent of morphospace exploitation is not reconstructed for this time slice, our CPMS shows that the majority of the morphological divergence had occurred by this time. This complements a previous observation by Werdelin [8] of a separation in morphospace occupation by the small/medium- and large-bodied cats (*contra* [9]). Our CPMS indicates that this separation has a deep-rooted history and that skull shape evolution did indeed follow different trajectories in small/medium- (including *Puma concolor*) and large-bodied cats. We emphasize that shape may never be under direct selection forces, its evolution being a trade-off between selection on latent factors such as function, ecology/environment, or development.

Throughout cat evolution, machairodontine ancestral nodes are consistently reconstructed as being separate from contemporary feline nodes, implying that large cats as a whole (‘Panthera’ lineage and large machairodontines) appear to have distinct trajectories in morphospace occupation through time. It appears as though large ‘Panthera’ lineage taxa move into regions of morphospace that were previously occupied by machairodontines, which themselves continuously expand outwards in morphospace. Thus, there is a sequential filling of morphospace, first by machairodontine ancestors, then by ‘Panthera’ lineage cats. As a caveat, we consider the possibility that the full range of morphospace occupancy of the ‘Panthera’ lineage deeper in time is not fully reconstructed, because the positions of ancestors are reconstructed primarily from morphospace coordinates of younger taxa. Nevertheless, the method does take into account possible ancestral conditions, and it would be interesting to see how further fossil discoveries match the patterns inferred from internal nodal reconstructions.

In conclusion, despite their relatively recent origin and unique specializations, cats have experienced significant changes in cranial construction, exhibiting instances of convergence, development of ‘extreme’ morphologies, and disjoint spatial and temporal patterns of morphological space occupation. We use a popular animal group to highlight the thrust of large-scale trees [57] as an invaluable tool to quantify the dynamics of character changes, and we hope that this study will promote renewed interest in similar adaptive radiations, both among mammals and in other organisms.

## Supporting Information

Figure S1 **Loadings for the first four PC axes from the PCA of 29 size-adjusted cranial linear variables in 332 specimens of extant and fossil felids.** The loadings for the first four PC axes are shown as bar plots. Numbers correspond to the variables listed in the legend for Fig. 3.
(TIF)

Figure S2 **Two-dimensional morphospace delimited by the first two PC axes from the PCA of 29 size-adjusted cranial linear variables in 332 specimens of extant and fossil felids.** A specimen-level morphospace was built using PCA and the first two PC axes were plotted. Lineages are shown in different colours, except machairodontine lineages (‘Machairodus’, ‘Metailurus’, and ‘Smilodon’ lineages), which are all treated as a single group, Machairodontinae, in this plot. Numbers correspond to MorphID in Table S1.
(TIF)

Figure S3 **Two-dimensional morphospace delimited by PC1 and PC3 from the PCA of 29 size-adjusted cranial linear variables in 332 specimens of extant and fossil felids.**
(TIF)

Figure S4 **Two-dimensional morphospace delimited by PC1 and PC4 from the PCA of 29 size-adjusted cranial linear variables in 332 specimens of extant and fossil felids.** Note machairodontine taxa separating out in morphospace from feline taxa.
(TIF)

Figure S5 **A one-dimensional chronophylomorphospace plot along PC1.** Transition of PC1 across phylogeny through time can be plotted following the methods of Sakamoto et al. [6] using maximum likelihood ancestor character estimation. The 95% confidence intervals of the ancestor estimates are shown as error bars. Node and branches are coloured according to monophyletic clade membership. Colours and numbers are as in Fig. 2.
(TIF)

Figure S6 **A one-dimensional chronophylomorphospace plot along PC2.** Transition of PC2 across phylogeny through time can be plotted following the methods of Sakamoto et al. [6] using maximum likelihood ancestor character estimation. The 95% confidence intervals of the ancestor estimates are shown as error bars. Node and branches are coloured according to monophyletic clade membership. Colours and numbers are as in Fig. 2.
(TIF)

Figure S7 **Tree of** **Fig. 2** **with branches scaled using an alternative method.** Branches were scaled by using first and last occurrence dates for all taxa (assuming fossil age ranges as known temporal distributions).
(TIF)

Figure S8 **Tree of** **Fig. 2** **with branches scaled using a third method.** Branches were scaled by taking midpoint dates for all taxa (assuming the modern time slice as the upper margin of error).
(TIF)

Figure S9 **Two-dimensional CPMS plot using the tree of Fig. S7.** A two-dimensional CPMS was plotted using the first two PC axes and a tree with branch lengths scaled according to the second method outlined in Text S2. Note that while the branching patterns of the extant taxa are not that different from those in Fig. 6 and Video S1, those for machairodontines are noticeably different. Particularly in that the branching events are bunched together in a narrower period of time resulting in very short internal branches, while each of the terminal branches are very long.
(TIF)

Table S1 Felid taxa for morphometric analysis. Lineage membership and identification numbers are listed for each taxon. MorphID, identification key for specimen-level morphometric plots in Supplementary Materials; PhyloID, identification key according to phylogeny and used throughout the main text.
(XLS)

Table S2 Notes on taxa used in cladistic analysis. The specimens observed for each OTU along with other relevant information such as locality or age are noted.
(XLS)

Table S3 Data matrix for cladistic analysis. Character numbers (columns) correspond to character descriptions given in Text S1. The supraspecific OTU, Felinae, is scored using the five feline taxa, *Felis silvestris*, *Leopardus pardalis*, *Puma yagouaroundi*, *Panthera leo*, and *Panthera tigris*, according to the following scheme: Characters are scored such that if the sum of the five taxa for any given character  = 0, then score for FELINAE  = 0; if sum  = 1, then FELINAE  = 0; if sum  = 2, then FELINAE  =  [0 1]; if sum  = 3, then FELINAE  =  [0 1]; if sum  = 4, then FELINAE  = 1; and if sum  = 5, then FELINAE  = 1. The above scheme is suitable for binary character scorings (0 or 1), so excludes character 18 in which 4 out of 5 taxa are scored 2, thus FELINAE  = 2. Character scores for each feline taxa are given separately from the main character matrix. Matrix is formatted for TNT [25].
(XLS)

Table S4 First and last occurrences of extant taxa, and mean age of fossil species, all expressed in millions of years. These dates were used to scale the branches of the composite phylogeny (Fig. 2). Mean ages for the fossil taxa were used, but see Text S2 for alternative methods and their effects on branch lengths.
(XLS)

Table S5 Raw variables. The raw measurements of the 29 variables are shown for each specimen.
(XLS)

Table S6 Mosimann’s transformed variables. The 29 variables (Table S5) were adjusted for size through Mosimann transformation for each specimen.
(XLS)

Table S7 Cross tabulation of prior classifications and predictions in LDA for each lineage. The rows are the prior classifications while columns are predicted lineage classifications. The values within each cell are the numbers of specimens in a given row (prior classification) assigned the classifications according to the columns, with correct classifications in blue and incorrect classifications in pink. For instance, the total number of specimens in the Bay Cat lineage is 13 but only 9 have been correctly assigned to the Bay Cat lineage, while 3 have been classified as Caracal lineage and 1 as Puma lineage; the accuracy is the proportion 9/13 = 0.692.
(XLS)

Table S8 Prior classifications, predicted classifications, and posterior probabilities in LDA for each specimen. The prior classifications and classifications predicted by LDA is shown, with correct classifications in blue and incorrect classifications in pink. Posterior probabilities for each specimen are also shown.
(XLS)

Table S9 Scores of all specimens on all PC axes. The Mosimann transforms were subjected to PCA and the first 11 PC axes were used for subsequent analyses. The scores of each specimen along all 29 PC axes are presented here.
(XLS)

Table S10 NPMANOVA post-hoc pairwise comparisons between lineages using PC1–PC11. The upper off-diagonal cells are *F*-values while the lower off-diagonal cells are *p*-values with sequential Bonferroni significance. Significant *p*-values are highlighted in pink.
(XLS)

Text S1 List of characters used in phylogenetic analysis. The 44 characters along with their states are listed and described. The coded data matrix is in Table S3.
(DOC)

Text S2 Details on branch scaling and the effect of different choices in terminal ages on PCM results.
(DOC)

Video S1 Three-dimensional movie output of the chronophylomorphospace. This was generated using the new R function, chronoPTS2D, and outputted as a spinning movie through the rgl R library [49]. Fig. 6 is an annotated screen capture of this plot.
(GIF)
