# Supplementary figures and images for: Convergence and Divergence in the Evolution of Cat Skulls: Temporal and Spatial Patterns of Morphological Diversity
Source: PLoS One. 2012 Jul 6;7(7):e39752. doi: 10.1371/journal.pone.0039752 (PMC3391202; doi:10.1371/journal.pone.0039752)

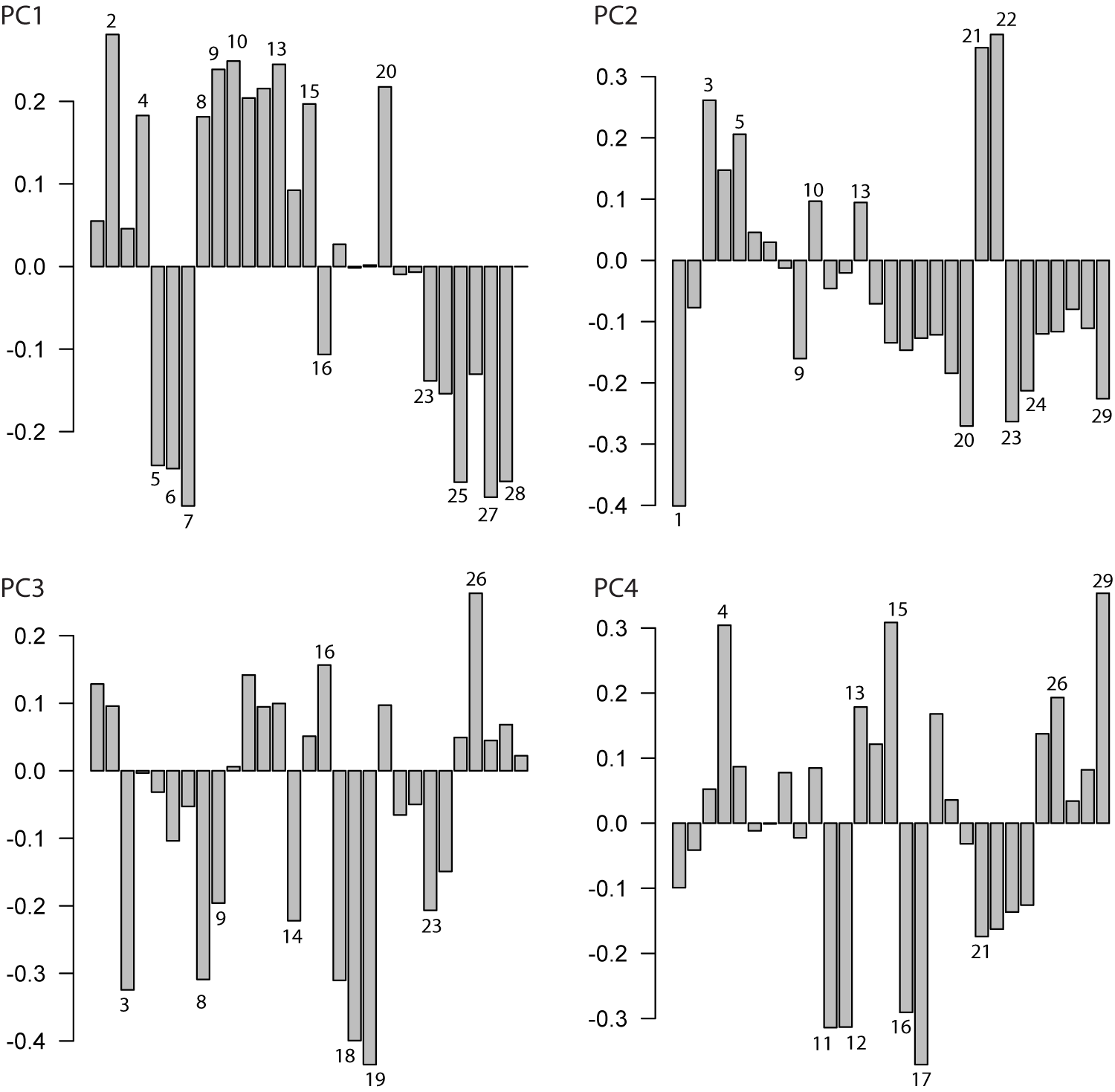

Supplement: Figure S1 — Loadings for the first four PC axes from the PCA of 29 size-adjusted cranial linear variables in 332 specimens of extant and fossil felids. The loadings for the first four PC axes are shown as bar plots. Numbers correspond to the variables listed in the legend for Fig. 3. (TIF) [file pone.0039752.s001.tif]

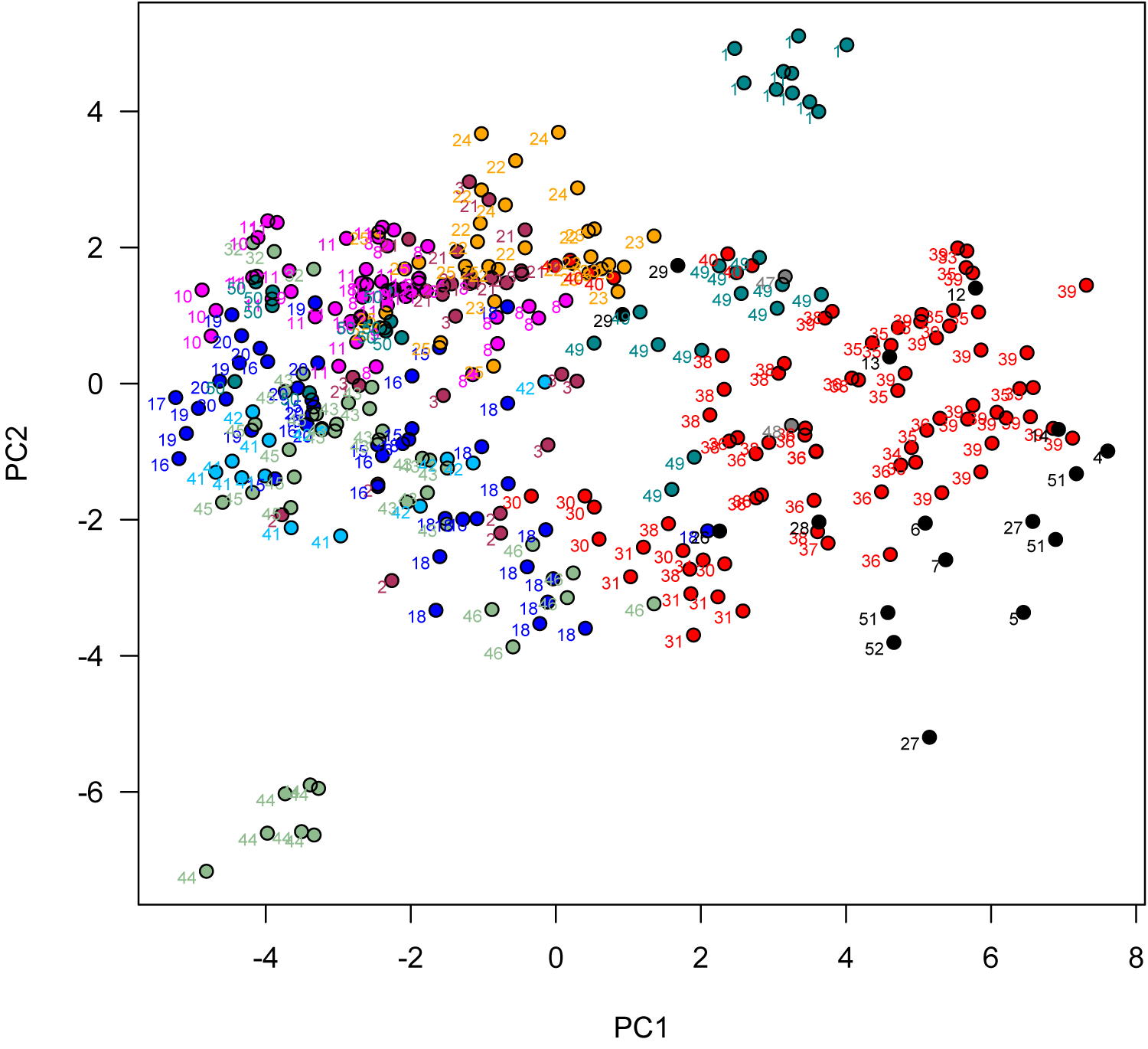

Supplement: Figure S2 — Two-dimensional morphospace delimited by the first two PC axes from the PCA of 29 size-adjusted cranial linear variables in 332 specimens of extant and fossil felids. A specimen-level morphospace was built using PCA and the first two PC axes were plotted. Lineages are shown in different colours, except machairodontine lineages (‘Machairodus’, ‘Metailurus’, and ‘Smilodon’ lineages), which are all treated as a single group, Machairodontinae, in this plot. Numbers correspond to MorphID in Table S1. (TIF) [file pone.0039752.s002.tif]

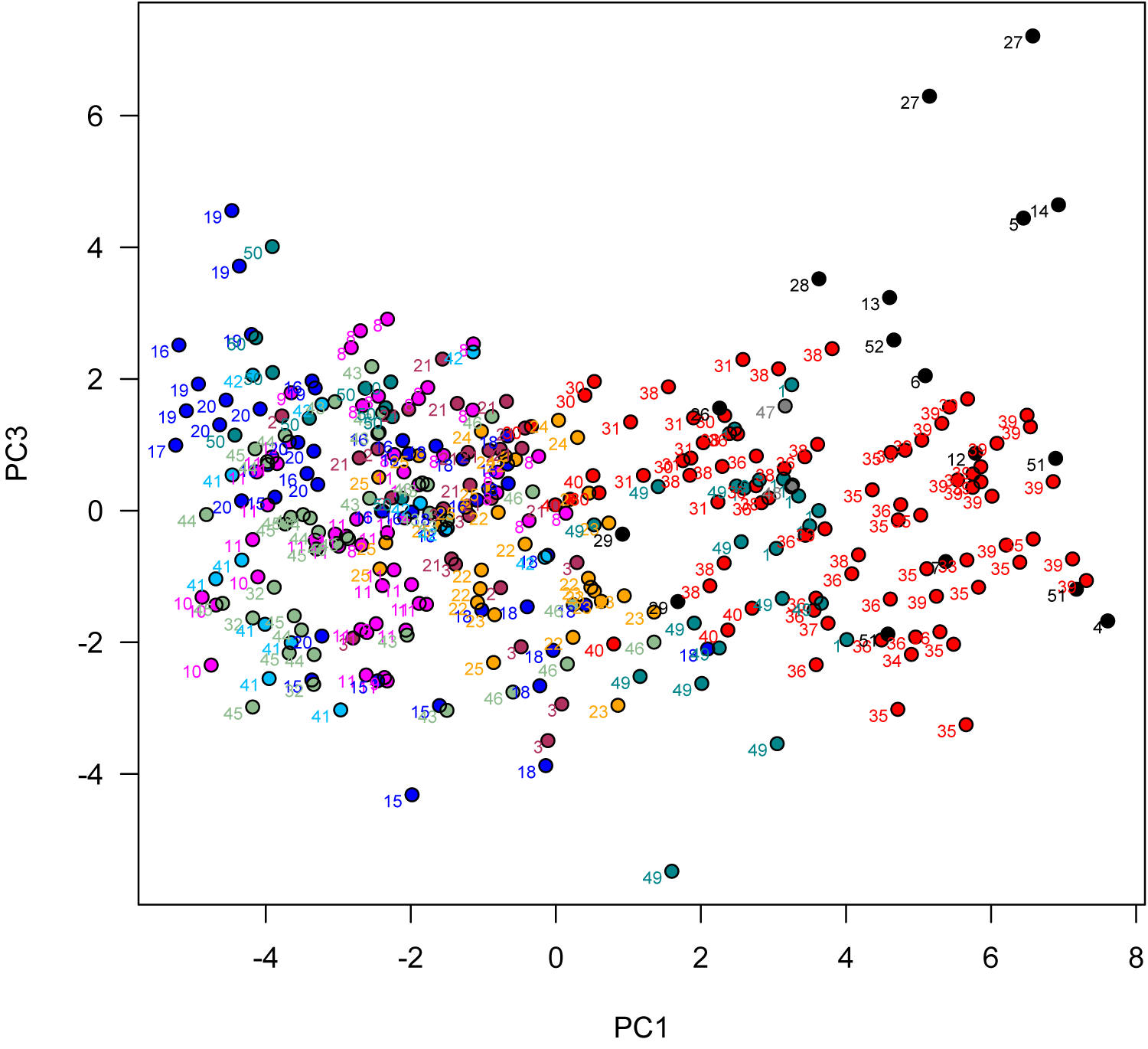

Supplement: Figure S3 — Two-dimensional morphospace delimited by PC1 and PC3 from the PCA of 29 size-adjusted cranial linear variables in 332 specimens of extant and fossil felids. (TIF) [file pone.0039752.s003.tif]

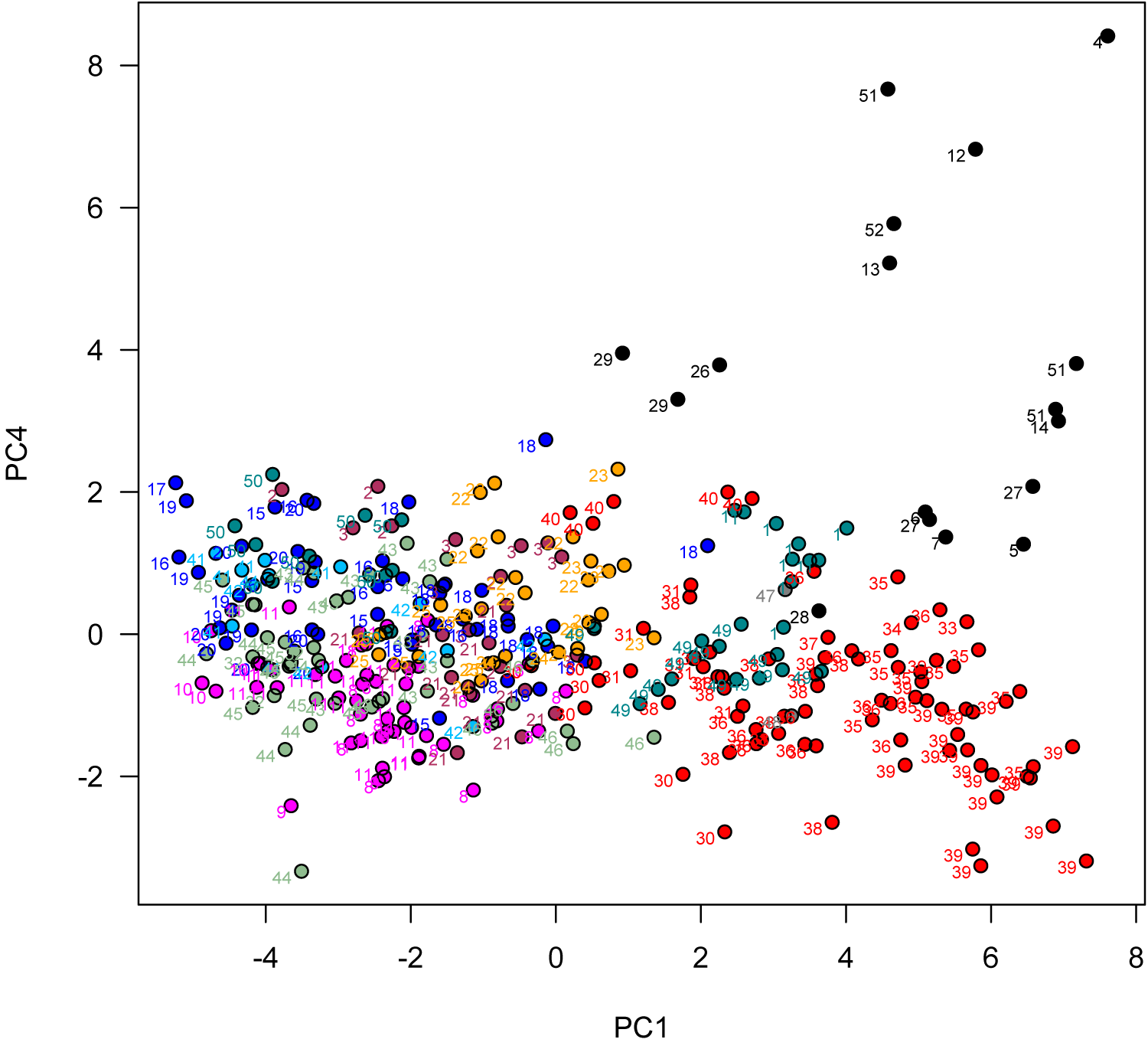

Supplement: Figure S4 — Two-dimensional morphospace delimited by PC1 and PC4 from the PCA of 29 size-adjusted cranial linear variables in 332 specimens of extant and fossil felids. Note machairodontine taxa separating out in morphospace from feline taxa. (TIF) [file pone.0039752.s004.tif]

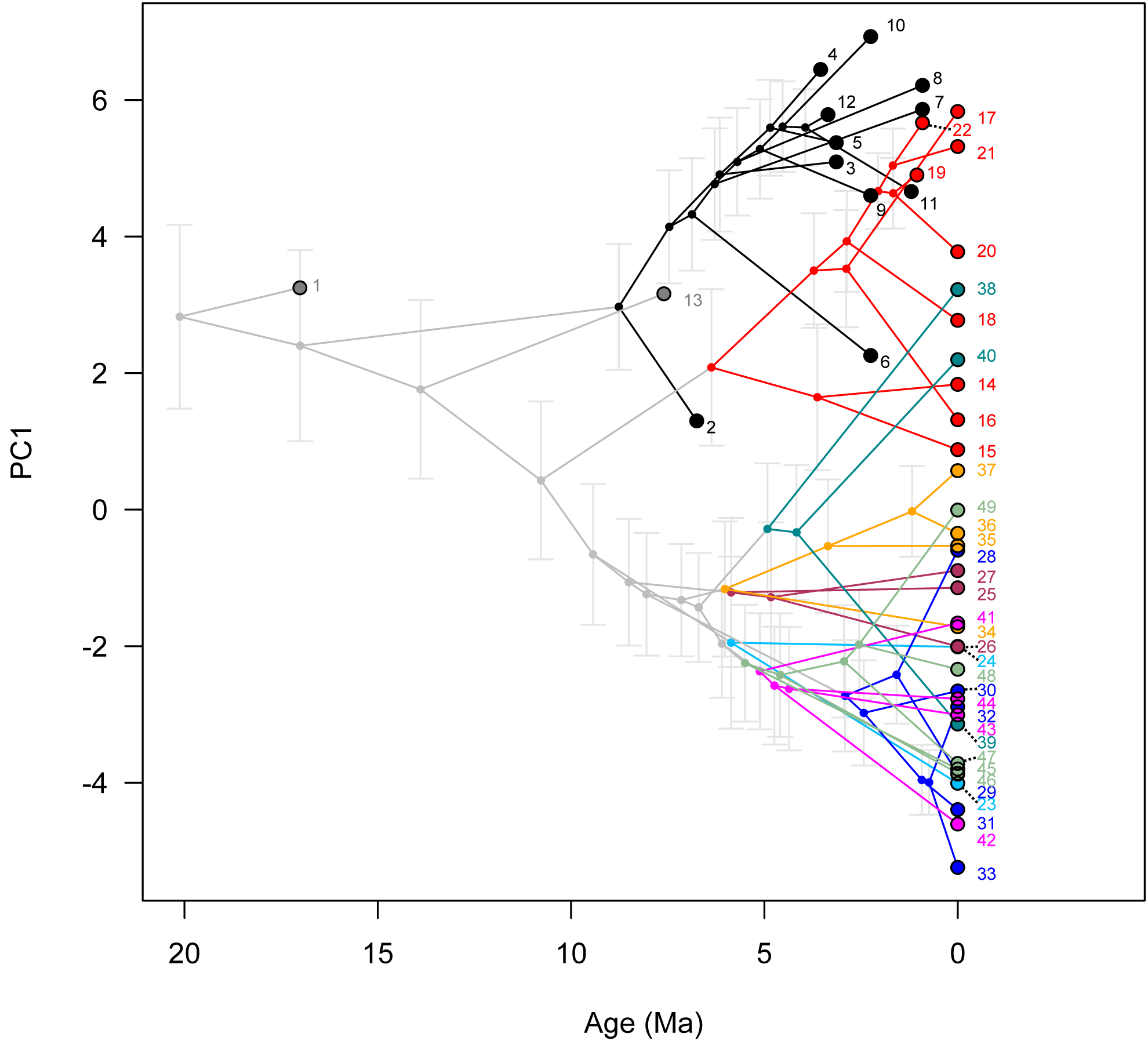

Supplement: Figure S5 — A one-dimensional chronophylomorphospace plot along PC1. Transition of PC1 across phylogeny through time can be plotted following the methods of Sakamoto et al. [6] using maximum likelihood ancestor character estimation. The 95% confidence intervals of the ancestor estimates are shown as error bars. Node and branches are coloured according to monophyletic clade membership. Colours and numbers are as in Fig. 2. (TIF) [file pone.0039752.s005.tif]

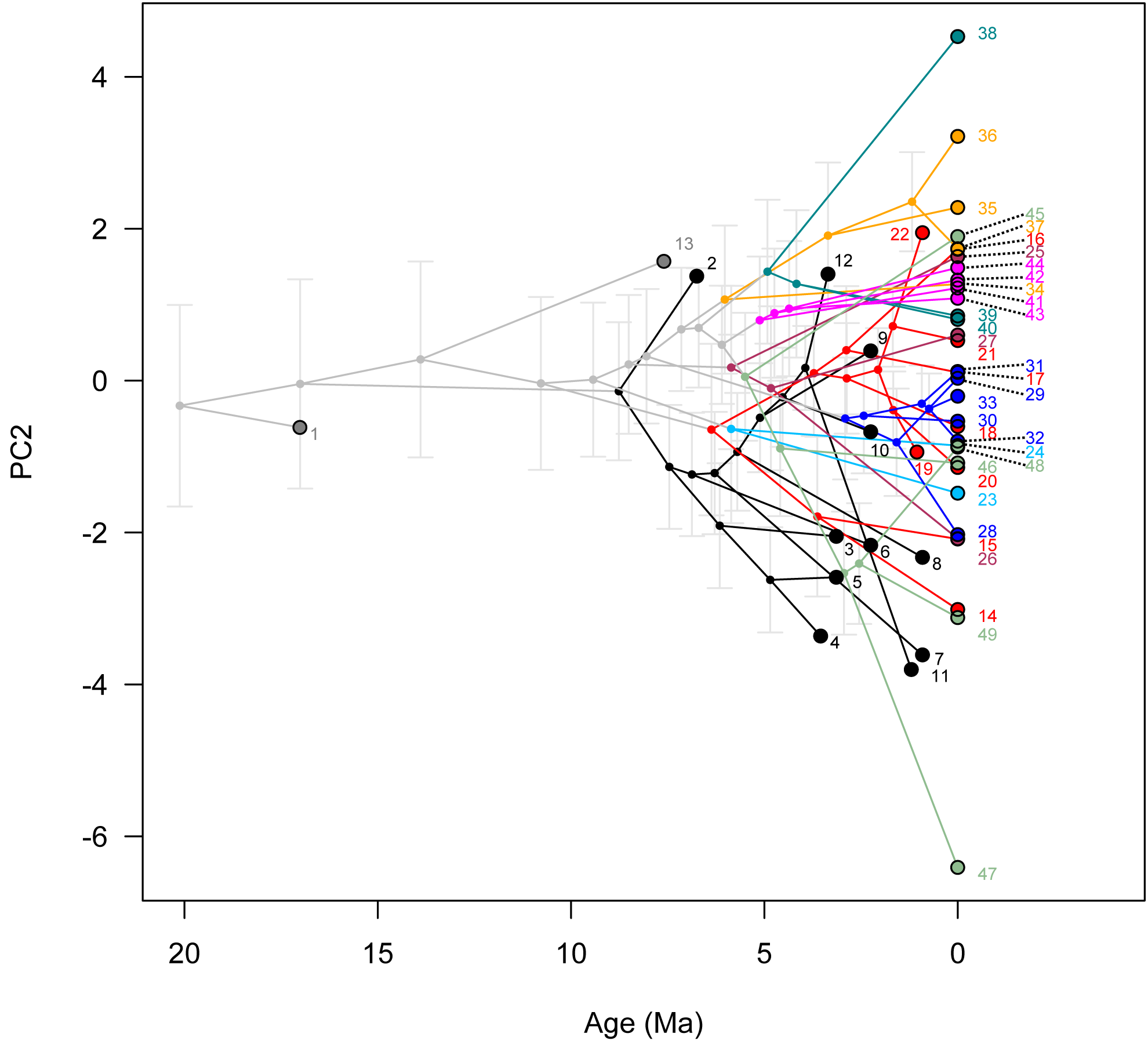

Supplement: Figure S6 — A one-dimensional chronophylomorphospace plot along PC2. Transition of PC2 across phylogeny through time can be plotted following the methods of Sakamoto et al. [6] using maximum likelihood ancestor character estimation. The 95% confidence intervals of the ancestor estimates are shown as error bars. Node and branches are coloured according to monophyletic clade membership. Colours and numbers are as in Fig. 2. (TIF) [file pone.0039752.s006.tif]

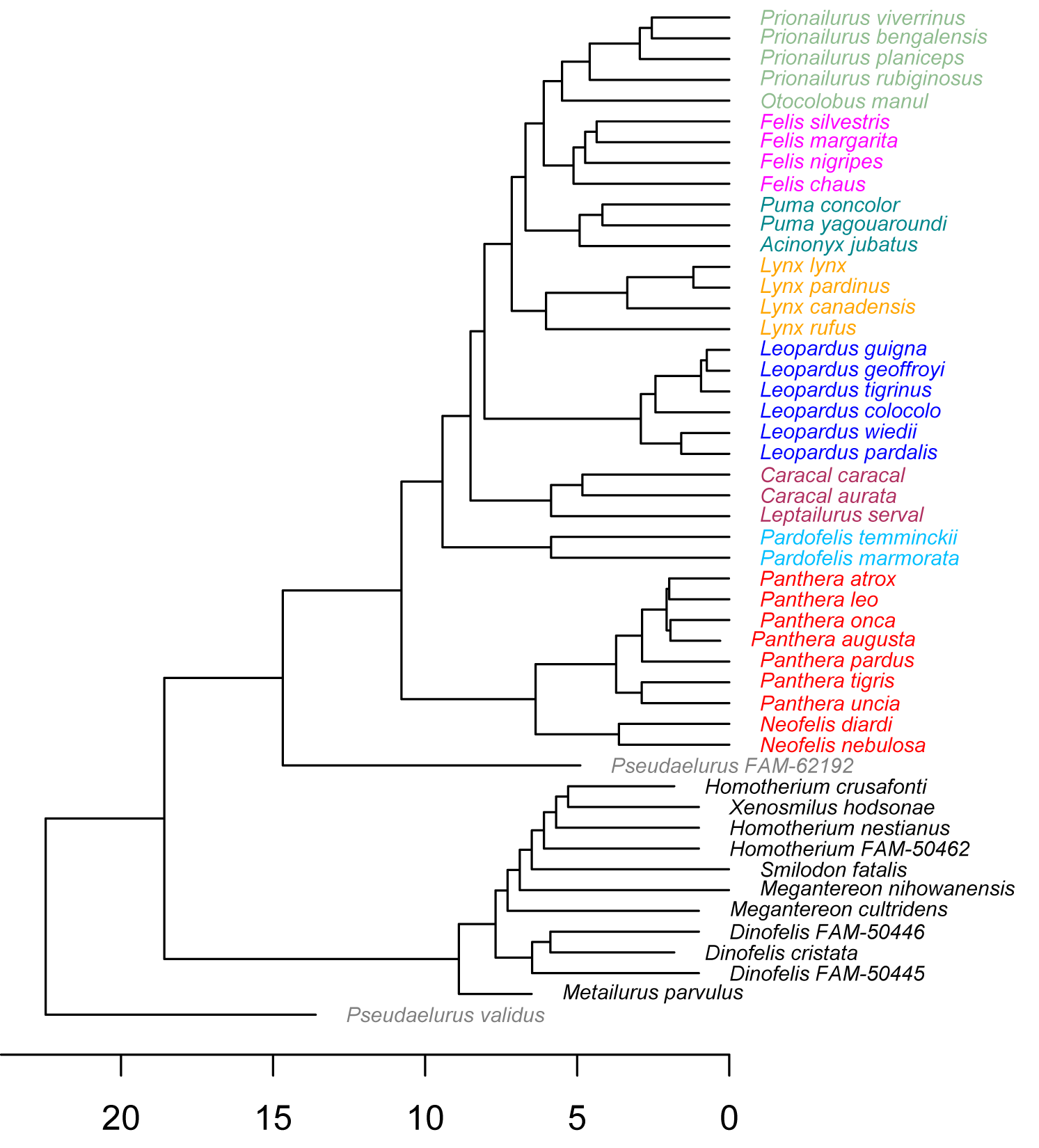

Supplement: Figure S7 — Tree of Fig. 2 with branches scaled using an alternative method. Branches were scaled by using first and last occurrence dates for all taxa (assuming fossil age ranges as known temporal distributions). (TIF) [file pone.0039752.s007.tif]

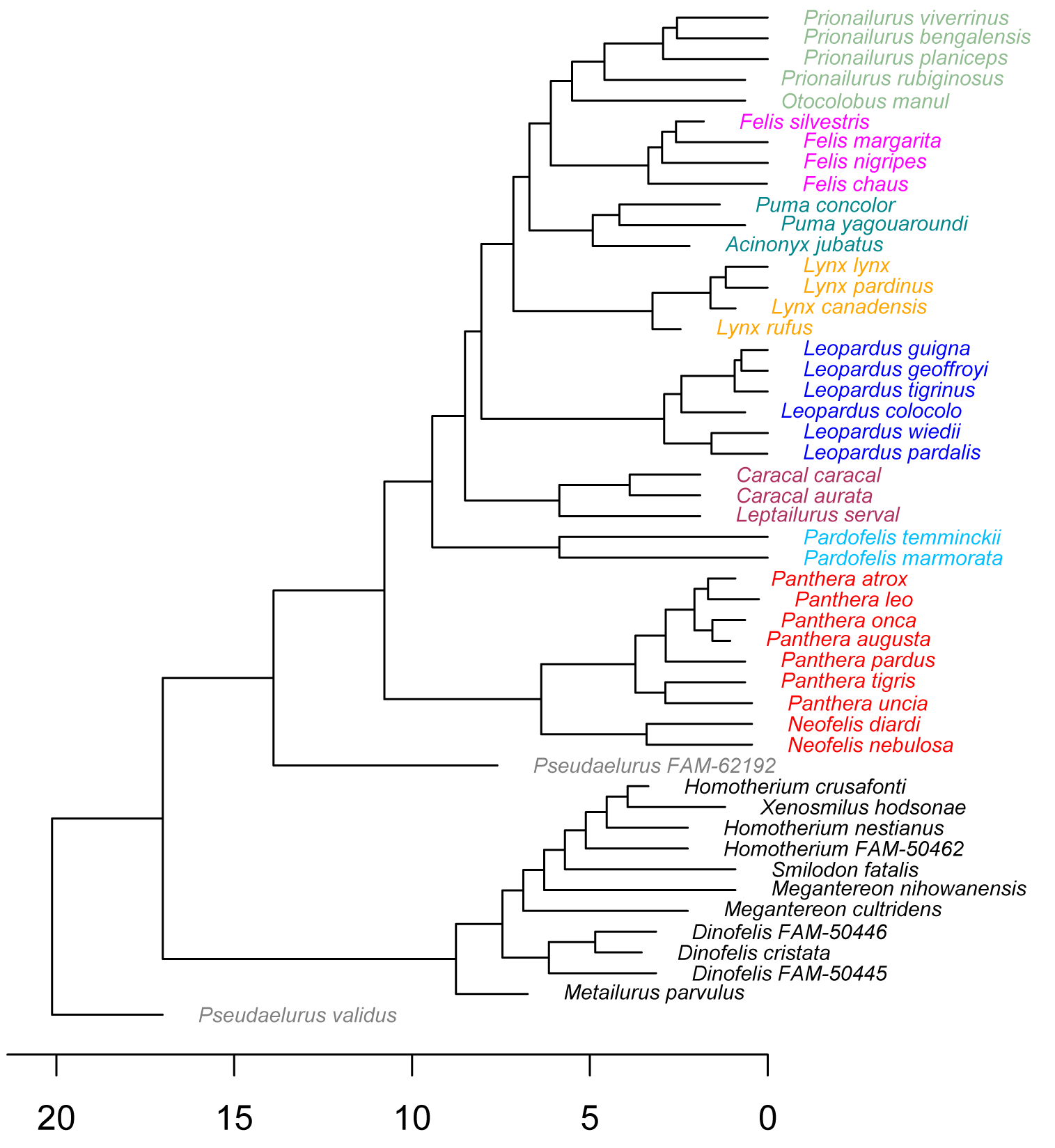

Supplement: Figure S8 — Tree of Fig. 2 with branches scaled using a third method. Branches were scaled by taking midpoint dates for all taxa (assuming the modern time slice as the upper margin of error). (TIF) [file pone.0039752.s008.tif]

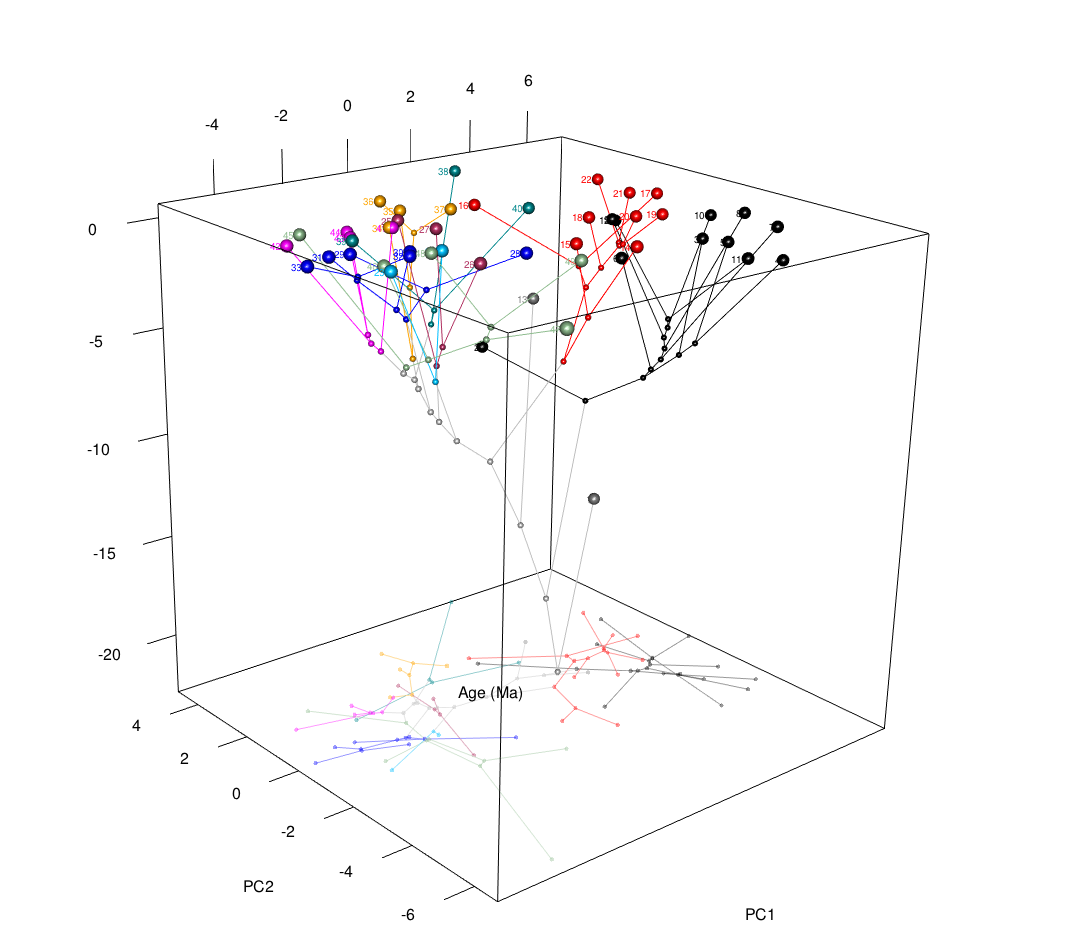

Supplement: Figure S9 — Two-dimensional CPMS plot using the tree of Fig. S7. A two-dimensional CPMS was plotted using the first two PC axes and a tree with branch lengths scaled according to the second method outlined in Text S2. Note that while the branching patterns of the extant taxa are not that different from those in Fig. 6 and Video S1, those for machairodontines are noticeably different. Particularly in that the branching events are bunched together in a narrower period of time resulting in very short internal branches, while each of the terminal branches are very long. (TIF) [file pone.0039752.s009.tif]

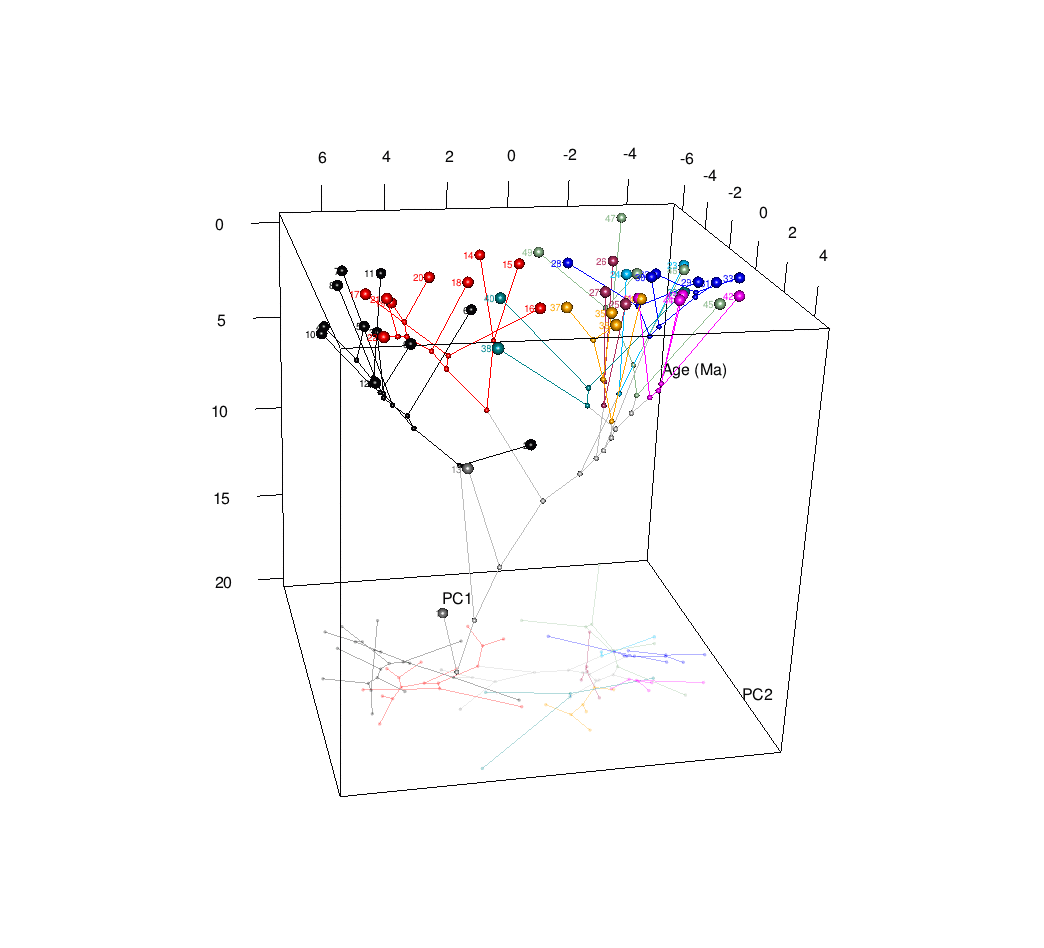

Supplement: Video S1 — Three-dimensional movie output of the chronophylomorphospace. This was generated using the new R function, chronoPTS2D, and outputted as a spinning movie through the rgl R library [49]. Fig. 6 is an annotated screen capture of this plot. (GIF) [file pone.0039752.s022.gif]
