# Supplementary material for: Convergence and Divergence in the Evolution of Cat Skulls: Temporal and Spatial Patterns of Morphological Diversity
Source: PLoS One. 2012 Jul 6;7(7):e39752. doi: 10.1371/journal.pone.0039752 (PMC3391202; doi:10.1371/journal.pone.0039752)
Supplement: Text S1 — List of characters used in phylogenetic analysis. The 44 characters along with their states are listed and described. The coded data matrix is in Table S3. (DOC) [file pone.0039752.s020.doc]

**SUPPORTING INFORMATION**

**Convergence and Divergence in the Evolution of Cat Skulls: Temporal and Spatial Patterns of Morphological Diversity**

Manabu Sakamoto & Marcello Ruta

**S1 Character list.** Numbers in parentheses correspond to the character numbers of Salesa et al. [24]. The data matrix is in Table S3. Notes and comments on specimens examined are in Table S2.

**Characters from Salesa et al. [24]:**

1. Upper canine crenulations (3): 0, absent; 1, faint; 2, prominent
2. Procumbent upper incisor arcade (5): 0, absent; 1, present
3. Presence of P1 (6): 0, present; 1, absent
4. Orientation of P3 and P4 (7): 0, aligned; 1, not aligned
5. Mesial cusp of P3 (8): 0, absent; 1, present
6. Disto-lingual basal expansion of the P3 crown (9): 0, present; 1, absent
7. Mesio-distal elongation of the P3 crown (10): 0, absent; 1, present
8. Position of P4 protocone (12): 0, at level of mesial border of parastyle; 1, distal to the parastyle
9. P4 ectostyle (13): 0, absent; 1, present
10. P4 parastyle (14): 0, smaller than or similarly sized to the protocone; 1, clearly larger than the protocone
11. M1 crown (15): 0, bucco-lingually elongated; 1, rounded
12. Mandibular symphysis outline (16): 0, gently curved; 1, nearly vertical
13. p2 (18): 0, present; 1, absent
14. p3 mesial cuspid (19): 0, absent; 1, present
15. p3 distal cuspid (20): 0, small; 1, well-developed
16. p4 mesial cuspid (21): 0, small; 1, well-developed
17. p4 distal cuspid (22): 0, small; 1, well-developed
18. m1 talonid (25): 0, present; 1, reduced; 2, absent

**New or amended characters:**

1. Caniniform upper incisors. Many machairodontine taxa show caniniform incisors, most prominent in *Homotherium*, *Xenosmilus*, and *Machairodus*. 0, incisors not caniniform; 1, intermediate in form between incisiform and caniniform; 2, incisors caniniform
2. Upper incisors enlarged. An enlargement of the upper incisors is a feature of derived machairodontines. While enlargement is usually associated with a caniniform morphology, the reverse is not true. 0, upper incisors not enlarged; 1, upper incisors enlarged
3. Medio-lateral compression of C1. Derived machairodontines exhibit extreme medio-lateral compression of the upper canines. However, many taxa have intermediate levels of compression between conical tooth cats and sabre-tooth cats. The compression was expressed as a ratio between the length and the width of C1 with states scored as follows: 0, ratio is less than 1.5; 1, ratio is greater than or equal to 1.5; 2, ratio is greater than or equal to 2 (C1 length is twice the width)
4. C1 keel. Felid canines typically have keels distally and lingually. However, derived machairodontines have a mesial (anterior) keel instead of a lingual keel, such that the canines have an anterior and posterior blade edge. 0, lingual keel; 1, mesial keel
5. Relative height of C1. A ratio between C1 height and C1 length was taken and states were scored as follows: 0, ratio is less than 2.5; 1, ratio is greater than or equal to 2.5; 2, ratio is greater than or equal to 3
6. Reduction of P2. A reduction in tooth count is typical to Felidae. The P2 is present in *Proailurus*, but is relatively reduced in many ‘*Pseudaelurus*’-grade taxa. In Machairodontinae, it is almost always either reduced or absent (complete lack of alveoli). In Felinae, P2 is either present or reduced (and very rarely absent). 0, present; 1, reduced; 2, absent (no alveoli)
7. Relative size of P3 to P4. In derived machairodontines P3 is substantially smaller in size compared to P4. A ratio between the length of P3 against the length of P4 was taken and states were scored as follows: 0, ratio is greater than 0.5; 1, ratio is less than or equal to 0.5 but greater than 0.3; 2, ratio is less than or equal to 0.3
8. Heavy wearing on the P4/m1 occlusal surface. While some older individuals of large felines show heavy wear on the carnassial blade surfaces, they are in no comparison to the extensive wearing seen in some machairodontines. The P4/m1 occlusal surface of these taxa displays a condition in which it resembles a self-sharpening pair of shearing blades. 0, absent to moderate wearing; 1, heavy wearing; 2, extensive wearing with self-sharpening blade edges
9. Procumbent lower incisor arcade. The lower incisors also form a procumbent arcade in some machairodontine taxa, namely *Xenosmilus* and *Homotherium* (but also *Machairodus*). 0, absent; 1, present
10. Caniniform lower incisors. The degree to which incisors are caniniform is not necessarily uniform across upper and lower incisors. Some taxa with clearly caniniform upper incisors do not have markedly caniniform lower incisors. 0, lower incisors not caniniform; 1, 1, intermediate in form between incisiform and caniniform; 2, lower incisors caniniform
11. Lower incisors enlarged. An enlargement of the lower incisors is observed in some derived machairodontines, most prominently in *Xenosmilus*, *Homotherium* and *Machairodus*. This feature is associated with an enlargement of the upper incisors but the reverse is not true. 0, lower incisors not enlarged; 1, lower incisors enlarged
12. Height of lower canines reduced. Derived machairodontines show a marked reduction in the height of the lower canines. A further reduction is seen in some taxa such that they are almost the same height as the enlarged incisors, and presumably functioned as an extra pair of caniniform incisors. 0, c1 not reduced; 1, c1 reduced
13. Presence of p3. The lower third premolar, p3, is generally present in most felids except some derived machairodontines: 0, present; 1, absent
14. Relative positions of p4 and m1. In derived machairodontines, the p4 is relatively laterally displaced in relation to the body of the ramus. This configuration places the p4 and m1 parallel to the midline of the skull, thus providing a shearing surface that is almost perfectly occluding with the upper carnassial (P4) blade edge. 0, p4 and m1 in line with the long axis of the mandibular ramus; 1, p4 is laterally displaced from the mandibular ramus and forming a shearing edge parallel to the skull midline
15. Nasal midline concave or convex. In felines, there is a marked concavity to the nasal midline, such that some taxa display a distinctly ‘m’ shaped profile in frontal view. In some machairodontines, the nasals do not form a concave profile towards the skull midline and instead either forms a flat or convex surface. 0, nasals concave towards midline; 1, nasals flat or convex towards midline
16. Shape of the naso-frontal suture (1, amended). In felines, the nasals taper posteriorly, so that the naso-frontal suture has a strong ‘V’-shape. In many machairodontines this tapering of the nasals is weaker such that the naso-frontal suture is more of a truncated shape. In some machairodontines, the nasals are very weakly tapered, and the naso-frontal suture is nearly right angled. 0, nasals strongly tapered posteriorly; 1, nasals weakly tapered posteriorly; 2, nasals very weakly tapered posteriorly
17. Lateral flexion of the dorsal process of the maxilla. In some felids, the dorsal process of the maxilla displays a strong lateral flexion. This is very prominent in *Panthera onca* among felines, and in *Smilodon* among machairodontines. 0, no lateral flexion of the dorsal process of the maxilla; 1, strong lateral flexion of the dorsal process of the maxilla
18. Mastoid process proportions. Derived machairodontines exhibit an enlargement and ventral elongation of the mastoid process. 0, mastoid process not enlarged; 1, mastoid process enlarged
19. Relative size of the sphenopalatal foramen. Some machairodontine taxa (but also *Panthera leo*, *P. atrox*, *P. spelaea*, and *P. palaeosinensis*) have relatively large sphenopalatal foramina. This is not necessarily size-associated since it is very small in *P. tigris* and similarly sized *P. augusta* (F:AM 69204). A ratio of the maximum diameter of the sphenopalatal foramen and the transverse diameter of the foramen magnum was taken, and scored into two states: 0, ratio is less than 0.5; 1, ratio is greater than or equal to 0.5
20. Relative position of the lacrimal foramen. The lacrimal foramen is either positioned on the orbital margin or tucked in behind the maxilla. These conditions are variable across Felidae as a whole. 0, lacrimal foramen on the orbital margin; 1, lacrimal foramen behind maxilla
21. Palatal ridge/groove. The palate in felids exhibits shallow grooves along the ventral surface running caudally from the incisive foramen. These are visually observable in ‘Panthera’ lineage taxa but substantially more obvious in machairodontine taxa. *Smilodon* and *Megantereon* display a further derived condition in which the medial portion of the palate is ventrally displaced from the main plane of the palate. 0, no visibly obvious groove or ridge (but may be felt by touch); 1, visibly obvious but shallow groove with ridges; 2, deep groove with distinctive ridges; 3, extreme medial ridges displacing medial palate ventrally from the main plane of the palate
22. Anterior compression of the auditory region. In machairodontines, the basicranium is rotated ventrally relative to the snout/palate, compressing the auditory region anteriorly. This anterior compression of the auditory region is most prominent in *Smilodon* and *Megantereon*. 0, no compression of the auditory region; 1, some compression of the auditory region; 2, very strong compression of the auditory region
23. Stylomastoid groove. Salles [58] observed several conditions in the groove for the stylomastoid foramen which is a faint (and sometimes difficult to see) groove running anteriorly and ventrally from the stylomastoid foramen around the anterior margin of the mastoid process. In felines this groove is clearly derived from the stylomastoid foramen (which is lateral to the tympanohyal pit). However, in most machairodontine taxa examined (and in *Hyperailurictis validus*), this groove originates from the more medial of the two foramina, the tympanohyal pit, instead of the stylomastoid foramen. 0, groove originating from the stylomastoid foramen; 1, groove originating from the tympanohyal pit
24. Mandibular process ventrally displaced. In most felids the mandibular process of the temporal bone is along the same plane as the basicranial surface. In some machairodontines however, the mandibular process is ventrally displaced away from the basicranial surface. This is probably in tandem with the ventral rotation of the palate in relation to the basicranial region and the ventral displacement of the mandibular process ensures that the jaw condyle is on the same (or proximal) functional plane as the palate/tooth row. 0, mandibular process on the same plane as the basicranial surface; 1, mandibular process ventrally displaced; 2, extreme ventral displacement of the mandibular process
25. Infracanine flange of the mandible (17, amended). Derived machairodontines display a mandibular flange ventral to the canines. The development of this flange varies. 0, no flange; 1, noticeable but relatively small flange; 2, prominent flange
26. Reduction of the coronoid process. 0, no reduction of the coronoid process; 1, moderate reduction of the coronoid process; 2, strong reduction of the coronoid process (almost reduced to a small stump)

**Literature cited in supporting information**

58. Salles LO (1992) Felid phylogenetics: extant taxa and skull morphology (Felidae, Aeluroidea). *American Museum Novitates* **3047**: 1-67.
