# Supplementary material for: Convergence and Divergence in the Evolution of Cat Skulls: Temporal and Spatial Patterns of Morphological Diversity
Source: PLoS One. 2012 Jul 6;7(7):e39752. doi: 10.1371/journal.pone.0039752 (PMC3391202; doi:10.1371/journal.pone.0039752)
Supplement: Text S2 — Details on branch scaling and the effect of different choices in terminal ages on PCM results. (DOC) [file pone.0039752.s021.doc]

**SUPPORTING INFORMATION**

**Convergence and Divergence in the Evolution of Cat Skulls: Temporal and Spatial Patterns of Morphological Diversity**

Manabu Sakamoto & Marcello Ruta

**S2 Branch scaling and the effect of different choices in terminal ages on PCM results.** Branches can be scaled in a number of different ways but we follow the protocols outlined in [6] and use a combination of molecular divergence dates and fossil occurrence dates. Ideally, the fossil range of each taxon should be used, but that is rarely known; the fossil record of several extant taxa is not known, and the dating of fossil-bearing strata may be inaccurate or be accompanied by large margins of uncertainties. In the case of extant taxa, regardless of their uncertain fossil ranges, we do know their upper temporal range, i.e., the present. Thus, their branches need to be extended to the present time to account for the amount of time elapsed since the divergences from their nearest relatives or hypothetical ancestors; Sakamoto et al. [6] dated the nodes according to molecular divergence dates and/or first occurrence dates, whichever is available or older, and simply extended the extant terminal branches to the modern time (0 Ma). However, in our case study, where we have a substantial amount of fossil taxa (most with uncertain temporal ranges), it may not be appropriate to use, respectively, the lower temporal range as the first occurrence date and the upper temporal range as the last occurrence date, because such ranges are error margins rather than definitive first and last occurrence dates. For instance, the three closely related (or even conspecific) *Dinofelis* OTUs are from geographically proximal localities (Shanxi and Henan Provinces, China) but the dating of their localities are so broad (i.e. 5.28 to 1 Ma) that if we were to take this range as the first and last occurrence dates, then these three OTUs will have had 4 Ma of independent evolution since their divergences from one another (see Fig. S7), which is rather unlikely given their extreme similarities (see results of cladistic analysis). Previous authors have used midpoint values of the date range as a tentative estimate of fossil dates for the purpose of branch length scaling [33, 34].

On the other hand, in the interest of consistency, it is also possible to treat the extant ranges (including the upper extant range) as uncertainties and use midpoint values in the same manner as in the fossil cases. This approach would ignore the true biodiversity of the present-day time slice. Thus, there are at least three methods that could be used to scale branches: 1) the method we took in the main analysis, i.e. a mixture of using first and last occurrence dates (extant) and midpoint dates (extinct); 2) using first and last occurrence dates for all taxa (assuming fossil age range as a known temporal distribution); and 3) taking midpoint dates for all taxa (assuming the modern time slice is the upper margin of error). We compare the effects these three branch lengths have on phylogenetic comparative tests (MPVR and *K*-test).

Using the two alternative trees (branch scaling methods 2 and 3 above; Figs S7 and S8 respectively), we conducted MPVR and *K*-tests in the identical manner as described in the main text for tree 1 (Fig. 2) using the appropriate number of phylogenetic PCO axes (cut-off at 95% variance) and species-mean PC scores of the first 11 PC axes for MPVR, and the first 4 PC axes for *K*-tests. Results are marginally different. The numbers of PCO axes that explain more than 95% of total phylogenetic variance in each tree are similar at 23 for tree1, 22 for tree 2, and 21 for tree 3. Using tree 2, MPVR detects significant and relatively strong phylogenetic signal in morphospace (*R*^2^ = 0.774; *p* = 1.72 × 10^-15^), compared to that of tree in Fig. 2 (*R*^2^ = 0.784; *p* = 3.25 × 10^-13^). Tree 3 also results in similar results (*R*^2^ = 0.762; *p* = 1.75 × 10^-12^). Blomberg’s *K*-test results are similar as well: for tree 2, *K*_PC1_ = 1.19 (*p* = 1 × 10^-4^), *K*_PC2_ = 0.371 (*p* = 1.2 × 10^-3^), *K*_PC3_ = 0.337 (*p* = 0.0155), *K*_PC4_ = 0.812 (*p* = 1 × 10^-4^); and for tree 3, *K*_PC1_ = 1.13 (*p* = 1 × 10^-4^), *K*_PC2_ = 0.289 (*p* = 8 × 10^-4^), *K*_PC3_ = 0.234 (*p* = 0.035), *K*_PC4_ = 0.752 (*p* = 1 × 10^-4^); as compared to *K*_PC1_ = 1.24 (*p* = 1 × 10^-4^), *K*_PC2_ = 0.305 (*p* = 5.5 × 10^-3^), *K*_PC3_ = 0.246 (*p* = 0.128), *K*_PC4_ = 0.809 (*p* = 1 × 10^-4^) for tree 1 used in the main analysis.

These results indicate that overall pattern in phylogenetic signal is only marginally affected by the different choices in ages. However, tree 2 does have an obvious impact on the way the CPMS plot looks (Fig. S9), and the multiple long branches in the Machairodontinae urge careful interpretation.

In conclusion, we have opted to use tree 1 for our phylogenetic comparative analyses and CPMS graphical representations due to its more realistic depiction of the modern biodiversity, treatment of error on fossil ranges, and graphical aesthetics of the resulting CPMS plots (i.e. if it looks wrong, then it probably is wrong), combined with the fact that tree selection have minimal effect on statistical results.
